# Building a neural network model to define DNA sequence specificity in V(D)J recombination

**DOI:** 10.1093/nar/gkaf551

**Published:** 2025-06-23

**Authors:** Justin C Harris, Jennifer N Byrum, Cooper B McKinney, Victoria Fairchild, Dee H Wu, Andrew H Fagg, Karla K Rodgers

**Affiliations:** Department of Biochemistry and Molecular Biology, University of Oklahoma Health Sciences, Oklahoma City, OK 73104, United States; Department of Microbiology and Immunology, University of Oklahoma Health Sciences, Oklahoma City, OK 73104, United States; Department of Microbiology and Immunology, University of Oklahoma Health Sciences, Oklahoma City, OK 73104, United States; Department of Microbiology and Immunology, University of Oklahoma Health Sciences, Oklahoma City, OK 73104, United States; Department of Radiological Sciences, University of Oklahoma Health Sciences, Oklahoma City, OK, United States 73104; Department of Computer Science, University of Oklahoma, Norman, OK 73019, United States; Department of Biochemistry and Molecular Biology, University of Oklahoma Health Sciences, Oklahoma City, OK 73104, United States; Department of Microbiology and Immunology, University of Oklahoma Health Sciences, Oklahoma City, OK 73104, United States

## Abstract

In developing lymphocytes, V(D)J recombination assembles functional antigen receptor (AgR) genes through rearrangement of the AgR loci to adjoin component gene segments. Each candidate gene segment for recombination is flanked by a recombination signal sequence (RSS), composed of heptamer and nonamer motifs separated by 12 or 23 base pairs. To initiate V(D)J recombination, the recombination activating proteins RAG1 and RAG2 create DNA double-stranded breaks between a 12/23-RSS pair and their adjoining gene segments. The basis for selection of individual RSSs during each V(D)J recombination event is not well understood due, in part, to the wide-spread distribution of the semi-conserved RSSs across the AgR loci. Using publicly-available data for V(D)J recombination efficiencies on randomized 12-RSSs, we first built a neural network model that delineates how changes in sequence at certain positions in the RSS affects recombination efficiency. Second, to interpret the model’s decision-making process, we repurposed the game theoretic SHapley Additive exPlanations (SHAP) approach, with the results illustrating how nucleotides at pairwise positions in the heptamer provide synergistic contributions to recombination efficiency. Third, we trained a nonamer-informed neural network model with varied nonamer RSS substrates, and subsequently identified interdependent effects between the heptamer and nonamer regions on recombination efficiency.

## Graphical Abstract

**Graphical Abstract:**
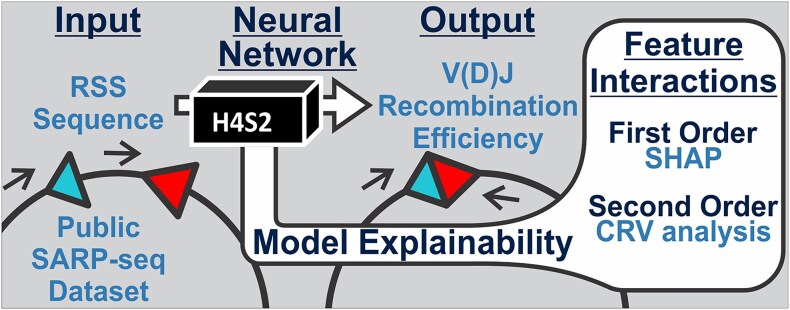

## Introduction

In the adaptive immune system, antigen receptors (AgRs), comprised of immunoglobulins (Igs) and T-cell receptors (TcRs), trigger immune responses upon recognition of antigens from disease-causing agents. The power of adaptive immunity arises from the vast sequence-diverse repertoires of AgRs, where virtually any pathogen-specific or tumor-specific antigen can be recognized by a particular Ig or TcR within a single organism's immune system [1]. The genetic capacity to generate these vast AgR repertoires originates during B- and T-cell development through V(D)J recombination [2–4]. This process rearranges AgR gene loci to join two-to-three gene segments (known as V, D, and J gene segments). AgR loci contain multiple versions of each of the gene segments, either all three types or only the V and J gene segments, which are widely dispersed across the unrearranged loci. Successful combinatorial joining of V-D-J or V-J gene segments generates the coding sequence for the antigen-binding region of functional AgRs. This process occurring collectively among B and T cells leads to the vast and highly diverse AgR repertoires in the adaptive immune system.

V, D, and J gene segments are flanked by recombination signal sequences (RSSs), rendering them as potential target candidates for recombination. Each RSS consists of a semi-conserved heptamer and a nonamer sequence separated by 12- or 23- nonconserved base pairs (bp), yielding two types of RSSs termed the 12-RSS and the 23-RSS [5]. Recombination only occurs between two gene segments that are flanked by RSSs of opposing spacer lengths, a restriction known as the 12/23 rule.

The V(D)J recombinase consists of a heterotetrametric RAG1 and RAG2 (RAG1/2) complex, which initiates the recombination reaction by recognizing and binding to a 12/23 RSS pair, and subsequently generating DNA double strand breaks (DSBs) between the RSS heptamer and its bordering gene segment [5, 6]. The RSS heptamer sequence is attributed to aiding in the DNA cleavage process; the nonamer sequence serves as an initial binding site for RAG1/2; and the 12/23 spacer regions align both the heptamer and nonamer regions within the RAG1/2 complex [2, 3, 6, 7]. Following DNA cleavage, DNA repair factors that function in nonhomologous end joining (NHEJ) join the AgR gene segments to generate the coding sequence of the AgR antigen binding domain [8, 9]. The NHEJ-mediated DNA repair reaction is imprecise with the addition or deletion of nucleotides from the coding joint, which further increases sequence diversity of the AgR repertoire. V(D)J recombination is essential for the maturation of B and T cells, as evidenced by the development of immune-related disorders caused by genetic defects of factors that function in either the DNA cleavage or joining steps of the recombination process [8, 10, 11]. Conversely, misregulation of when and where recombination can occur induces genomic instability resulting from RAG-mediated DNA DSBs at off-target sites that can lead to chromosomal deletions and translocations. Aberrant RAG-mediated recombination is associated with both generating the initial oncogenic event and producing driver mutations in the progression of oncogenesis [12–14].

There is an unequal utilization of gene segments by V(D)J recombination in forming the initial AgR repertoire in immature B and T cells, and the basis behind this unequal selection is a longstanding question. Gene segment selection is dependent, in part, on conformational changes in the gene loci to bring a 12- and 23-RSS pair into close proximity to the RAG1/2 complex [15–18]. Each of the AgR loci have unique structural and regulatory machinery that governs recombination [19, 20], while in contrast, the principles that define the binding interaction and cleavage propensity of RAG1/2 with the RSS substrates is common regardless of genetic context. For example, it is expected that the propensity of cleavage at a certain RSS pair will depend on DNA sequence-specificity as the RSSs vary in sequence. However, due to the length of the RSS and its sequence diversity, it has been challenging to define the rules that govern DNA cleavage propensity of the RSSs [21].

To undergo efficient V(D)J recombination, the RAG1/2 protein complex and the RSS’s DNA structures must work together to undergo major conformational changes, particularly within the two RSS heptamers [22, 23]. High resolution structures of the catalytically essential core RAG1/2 heterotetramer bound to a 12- and 23-RSS have been reported [22–24]. While these studies provided insight into the structures of the complex at differing stages of the DNA cleavage reaction, all of the solved structures only included RSSs with canonical heptamer and nonamer sequences. Consequently, it is not known how the structural behavior of variant RSS sequences through the RAG1/2 catalytic pathway may differ.

Numerous biochemical studies have investigated RAG1/2 binding and cleavage of select RSS substrates, collectively resulting in the analysis of RAG1/2 activity on hundreds of RSSs [2, 5, 12, 25–28]. However, as experiments were conducted using varying conditions and formats it has been difficult to compare results between different studies. Recently, we used a high-throughput assay, termed selective amplification of recombination products and sequencing (SARP-seq), to determine V(D)J recombination efficiencies for thousands of 12-RSS substrates that varied in the 3′ region of the heptamer and the adjacent 12 bp spacer region, resulting in the analysis of the largest set of potential RSS substrates in a single experimental assay [29]. As the RSS heptamer region undergoes large conformational changes during the RAG1/2 cleavage reaction [22], and the correlation between sequence and cleavage activity has not been well-defined, this region of the RSS is of particular interest. While the SARP-seq study showed a preference for alternating purine-pyrimidine (R-Y) base motifs in the 3′ portion of the heptamer and certain base preferences in the adjoining spacer region [29], it was challenging to identify the characteristics of favorable RSSs through a manual inspection of the activity level on each RSS substrate.

**Figure 1.**
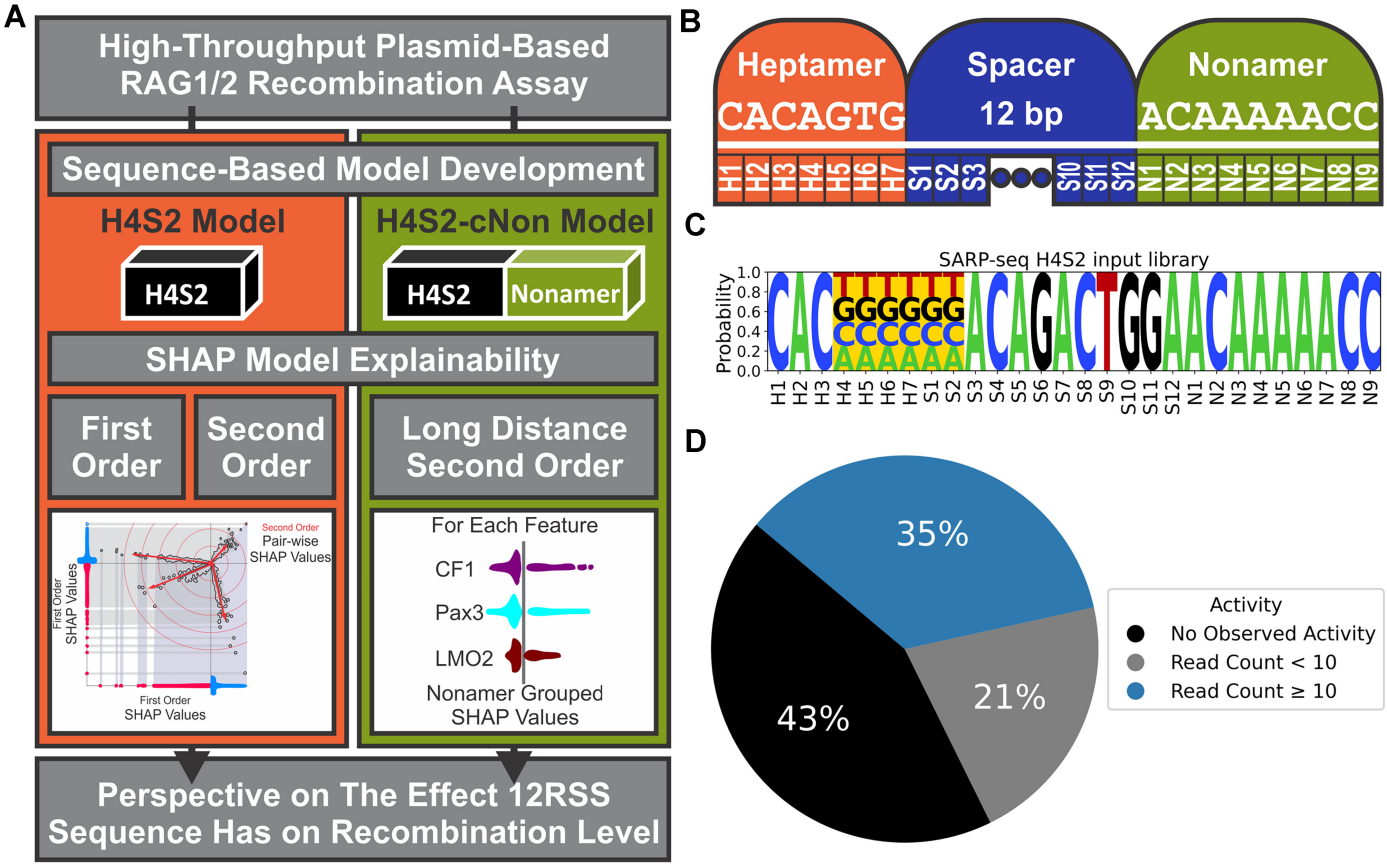
(**A**) Flowchart illustrating an overview of the steps taken throughout this study. (**B**) Diagram of canonical 12-RSS DNA sequence (above white line) and nomenclature of nucleotide position (below white line). The canonical 23-RSS heptamer and nonamer DNA sequence is the same as the 12-RSS but separated by 23 bp. (**C**) Sequence logo of the partially randomized N(H4-S2) 12-RSS substrate used in the SARP-seq experiment. (**D**) High, low, or no read counts of the 12-RSSs in the complete iSeq1 N(H4-S2) input dataset.

We propose that the initial SARP-seq experimental results provide deeper information on the contribution of pairwise base interactions to RAG1/2 activity. As machine learning is capable of identifying and utilizing highly complex and hidden interactions between input features, we built a sequence-based neural network model using the SARP-seq recombination data and RSS sequence as feature inputs to capture the impacts of the critical heptamer/spacer region of the RSS on V(D)J recombination efficiencies. For example, we demonstrate that the predicted V(D)J recombination levels can be negatively correlated with thermal stability of RSSs that differ in the H4-S2 region. Then using explainable artificial intelligence (AI) techniques, first- and second-order interactions between nucleotide positions were identified, which provides a granular understanding on how the heptamer/spacer region of the 12-RSS may promote or hinder V(D)J recombination efficiencies (shown as flowchart in Fig. 1A). A first-order interaction considers only one feature while marginalizing the effects of all other features. A second-order interaction considers a pair of features while marginalizing the rest of the features’ effects.

Further, we built a separate neural network model using experimental SARP-seq data that included the RSS heptamer/spacer region and three unique nonamers. Two of the three nonamers belong to cryptic RSSs, which are found outside the AgR loci and are involved in aberrant recombination events [21]. Using explainable AI, the nonamer-informed model revealed a largely modular behavior between the heptamer’s and nonamer’s effects on V(D)J recombination efficiencies, along with a few small identifiable long distance second-order interactions. Altogether, the trained models and the model explanations provide a previously unobtained resolution on the first and second-order interactions between key positions in the RSS sequence and their effect on V(D)J recombination efficiency.

## Materials and methods

Neural network models were built and trained using the experimental data obtained from a high-throughput recombination assay (SARP-seq). The models will predict V(D)J recombination efficiencies given a variant RSS sequence. Neural networks are trained mathematical representations that transform data inputs into a prediction output. The Supplementary Methods contains a detailed explanation of how neural networks were trained to utilize input features to calculate an output target (Supplementary Fig. S1). Beyond our introduction to building neural networks in Supplementary Methods, detailed aspects of machine learning have been previously reviewed [30–38]. The input datasets and the code to generate and analyze the models are available through a Zenodo Repository (https://doi.org/10.5281/zenodo.14903647) and a GitHub Repository (https://github.com/A-Blue-Jay/Building-a-Neural-Network-Models-Notebooks).

### SARP-seq N(H4-S2) dataset acquisition

Input data to build a neural network model, which we refer to as the H4S2 model, consisted of previously published results from a high-throughput V(D)J recombination assay, known as SARP-seq [29] (Supplementary Fig. S2A). The SARP-seq experiments quantified the V(D)J recombination efficiencies of 4096 plasmid substrates, where each substrate contained a different 12-RSS paired with a canonical 23-RSS. In the 12-RSSs of the plasmid library, all possible sequences within the four 3′ heptamer positions (H4-H7) and the adjacent first two base pairs (S1-S2) of the 12 bp spacer were represented, while the rest of the spacer (S3-S12) and the nonamer (N1-N9) were held constant (Fig. 1B and C). The plasmid substrate library, referred to here as N(H4-S2), was subjected to V(D)J recombination in mammalian cells, and the relative levels of recombination for each of the different 12-RSS-containing substrates evaluated using next generation sequencing (NGS) (Supplementary Fig. S2A). SARP-seq datasets lists 12-RSSs versus read count values obtained from NGS, where the read count is an indirect measure of V(D)J recombination activity on a given 12-RSS sequence [29].

Here, the input for both optimization of the k-models and training of the H4S2 model was obtained from the experimental replicate termed the iSeq1 dataset (in Hoolehan *et al.* [29]). RSSs that were absent in the iSeq1 dataset due to lack of detectable recombination activity were reintroduced with zero read counts, such that all 4096 RSSs were included prior to stratified k-fold generation, with the complete iSeq1 dataset listed in Supplementary Dataset S1 (Fig. 1D).

### Building the H4S2 model

**Figure 2.**
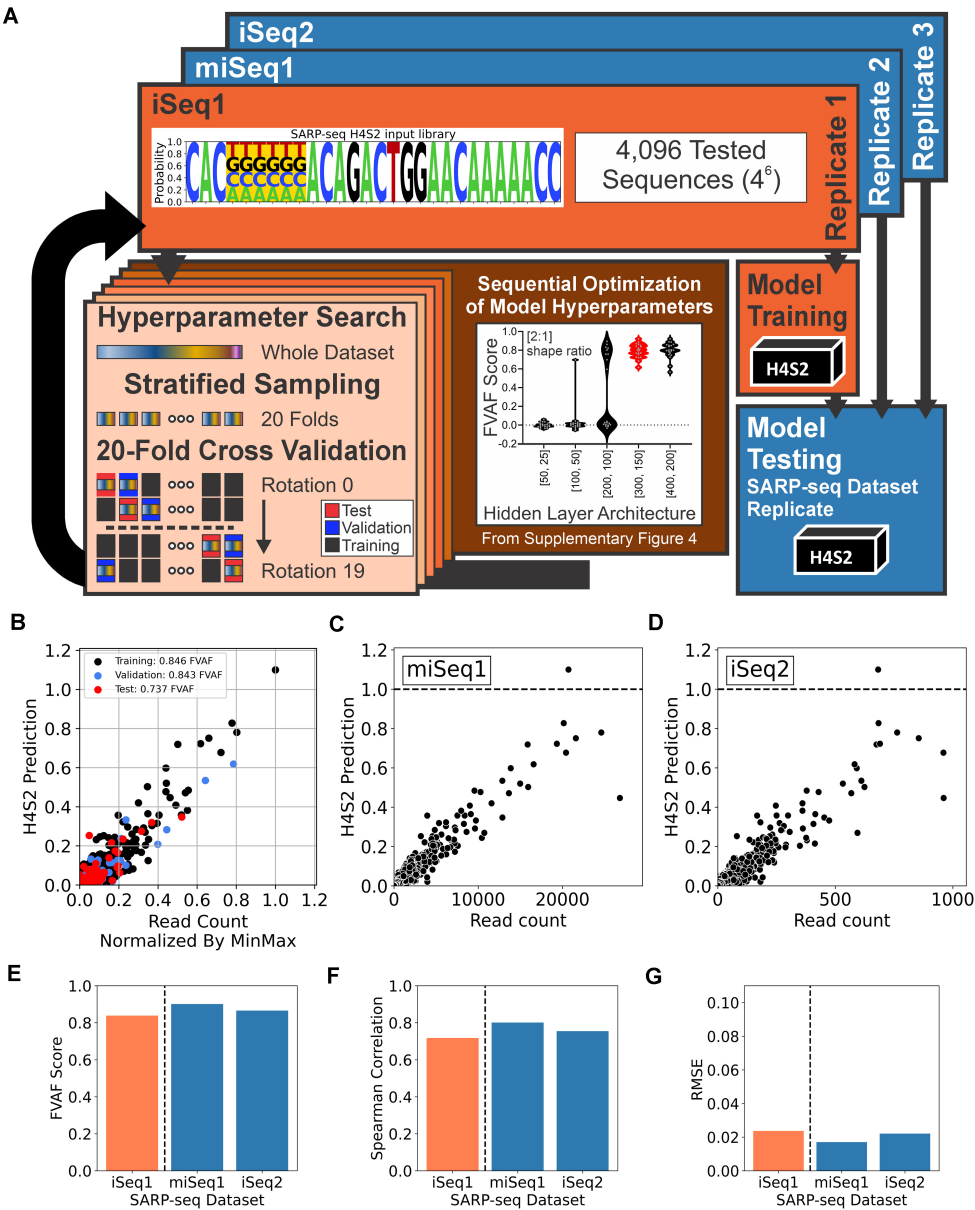
H4S2 model development and testing. (**A**) Flowchart illustrating H4S2 model development, training, and testing. Through multiple rounds of *k*-fold cross validation, each round attempted to optimize one hyperparameter and stratified sampling ensured a consistent distribution of input data for each of the 20 models. The optimized hyperparameters were used to train the H4S2 model. (**B**) Predictions of the training, validation, and test datasets from H4S2 model training. (**C** and **D**) H4S2 model predictions of the SARP-seq N(H4-S2) dataset’s experimental replicate miSeq1 (in panel C) and iSeq2 (in panel D). (**E**) FVAF score (${\mathrm{FVAF}} = 1 - ({\mathrm{Mean\;Square\;Error\;}}/\;{\mathrm{Variance)}}$; where $1 \ge {\mathrm{FVAF}} > - \infty )$, (**F**) Spearman-rank order correlation, and (**G**) RMSE for SARP-seq N(H4-S2) datasets’ training experimental replicate; iSeq1 (left bar in orange) and testing experimental replicates miSeq1 and iSeq2 (middle and right bars in blue).

The Keras (https://keras.io), SciKit-Learn [39], SciPy [40], and TensorFlow libraries [41] were used to construct, train, and optimize the H4S2 neural network model’s performance. A detailed overview of the steps to build the neural network model is provided in Supplementary Methods. In brief, one-hot encoding was used to transform the H4-S2 sequence in the 12-RSS into binary inputs, where each nucleotide base (A, T, G, C) was converted into a 4-bit representation for each position with each bit used as an input feature (Supplementary Fig. S1A). Stratified *k*-fold cross-validation, using the complete iSeq1 dataset, was performed to optimize the model’s encodings, architecture, and hyperparameters in a stepwise manner, as described in the following paragraph (Fig. 2A). A two-layer dense neural network was selected through multiple rounds of 20-fold cross-validation and evaluating on the training and validation sets. The DNA sequences were one-hot encoded into binary representations to predict the V(D)J recombination efficiencies based on the DNA sequence alone (Supplementary Fig. S3A and B). Additionally, early-stopping and dropout regularization were applied to prevent overfitting. The 20 models’ prediction targets were the dataset-wide normalized read count values for each 12-RSS in the complete iSeq1 dataset (Supplementary Dataset S1).

The distribution of the complete iSeq1 dataset was split into bins to allow for the use of stratified sampling (Supplementary Fig. S3C). The StratifiedKFold module from SciKit-Learn library split the complete iSeq1 dataset’s distribution into 20-fold (Supplementary Fig. S3D), each sharing the same asymmetric distribution as the original dataset. Next, 20 different rotations of data were created by rotating through the 20-fold, where each model had a different combination of 18-fold for training, one fold for validation and one fold for testing. Of the 20 rotations, each fold is used only once as a validation dataset and once for a different model’s testing dataset. A two-sample Kolmogorov–Smirnov (KS) test metric comparing each rotation and their training, validation, and testing subsets to the complete dataset indicated small differences between the read count distributions. The high *P*-values (of nearly 1.0) further support the KS test’s null hypothesis that each of the 20 rotations share a distribution with the same shape and scaling to the complete iSeq1 dataset’s distribution (Supplementary Fig. S3E). This consistency enabled the comparison of the 20 models that had been trained under differing hyperparameters. Each of the 20 models were trained for 100 epochs, halting when early-stopping regularization detected overfitting by monitoring the model's mean square error of the validation subset (Supplementary Fig. S3F). The training and validation subsets and the 20 model’s fraction of variance accounted for (FVAF) performance were used to guide the stepwise hyperparameters selection process. To confirm that the 20 models were optimized, a final exploration of the selected hyperparameters and architecture was performed (Supplementary Fig. S4A–F).

Using the optimized hyperparameters and architecture obtained from the *k*-fold cross-validation procedure above, a final H4S2 model was trained on the complete, min–max normalized iSeq1 dataset, with the dataset split once into training, validation, and test subsets using stratified sampling (Fig. 2B and Supplementary Dataset S1). The trained H4S2 model was rigorously tested on experimental replicates for the SARP-seq N(H4-S2) library, which represented unseen data for the H4S2 model (Fig. 2C and D). The experimental replicates were the miSeq1 and iSeq2 datasets from Hoolehan *et al.* [29], where the entire datasets (including 12-RSSs with zero read counts) were min–max normalized (see Supplementary Dataset S1). The performance of the H4S2 model's prediction was evaluated using FVAF scores, Spearman Rank Order Correlations, and Root Mean Squared Error (RMSE) for the training and testing experimental replicate datasets (Fig. 2E–G). To determine the extent that the H4S2 model’s prediction is affected by the inherit variation in the SARP-seq experimental data, the residual error between the H4S2 model’s prediction and the iSeq1 experimental measure of recombination (the *y*-axes) was compared to the standard deviation of in the measure of recombination across the iSeq1, iSeq2, and miSeq1 experimental replicates (the *x-*axes) (Supplementary Fig. S5A–E).

### Building the nonamer-informed H4S2-cNon model

A separate neural network model, which we refer to as the H4S2-cNon model, was trained on SARP-seq experimental data of the recombination efficiencies of three plasmid substrate libraries (where the resulting datasets are referred to as CF1, Pax3, and LMO2 according to the nomenclature in Hoolehan *et al.* [29]), where each library differed in the 12-RSS nonamer sequence. Each of the libraries contained four fully randomized base pair positions at the 3′ end of the heptamer (H4-H7) and one partially randomized position (T or G nucleotides denoted as K) in the adjoining spacer (S2), which altogether we designate here as N(H4-H7)K(S2). The resulting SARP-seq datasets contained NGS-based recombination efficiencies for the three separate plasmid libraries (CF1, Pax3, and LMO2). Here, the recombination efficiencies of each nonamer’s experimental replicates were separately averaged, including two experimental replicates each for the LMO2 and Pax3 libraries, and three experimental replicates for the CF1 library (Supplementary Dataset S1). Dataset-wide min–max normalization was performed where the read count values in the combined CF1, Pax3, and LMO2 libraries were rescaled to a range between 0 and 1 (Supplementary Dataset S1).

The H4S2-cNon model used the same hyperparameters and architecture as the H4S2 model (Supplementary Fig. S6A and B), while expanding the input shape to include encoding features for positions N1-N3 and N8-N9 (positions denoted in Fig. 1B). The H4S2-cNon model was trained to predict the normalized dataset-wide read count values (Supplementary Fig. S6C–E), and its performance metrics were calculated as done with the H4S2 model. The H4S2-cNon model’s performance was visually compared to the H4S2 model’s performance on predicting the more diverse N(H4-H7)K(S2) SARP-seq dataset (Supplementary Fig. S6F–K). The H4S2-cNon model was used to explore differences in interactions between the heptamer and nonamer region of the 12-RSS.

### The RIC score calculation

**Figure 3.**
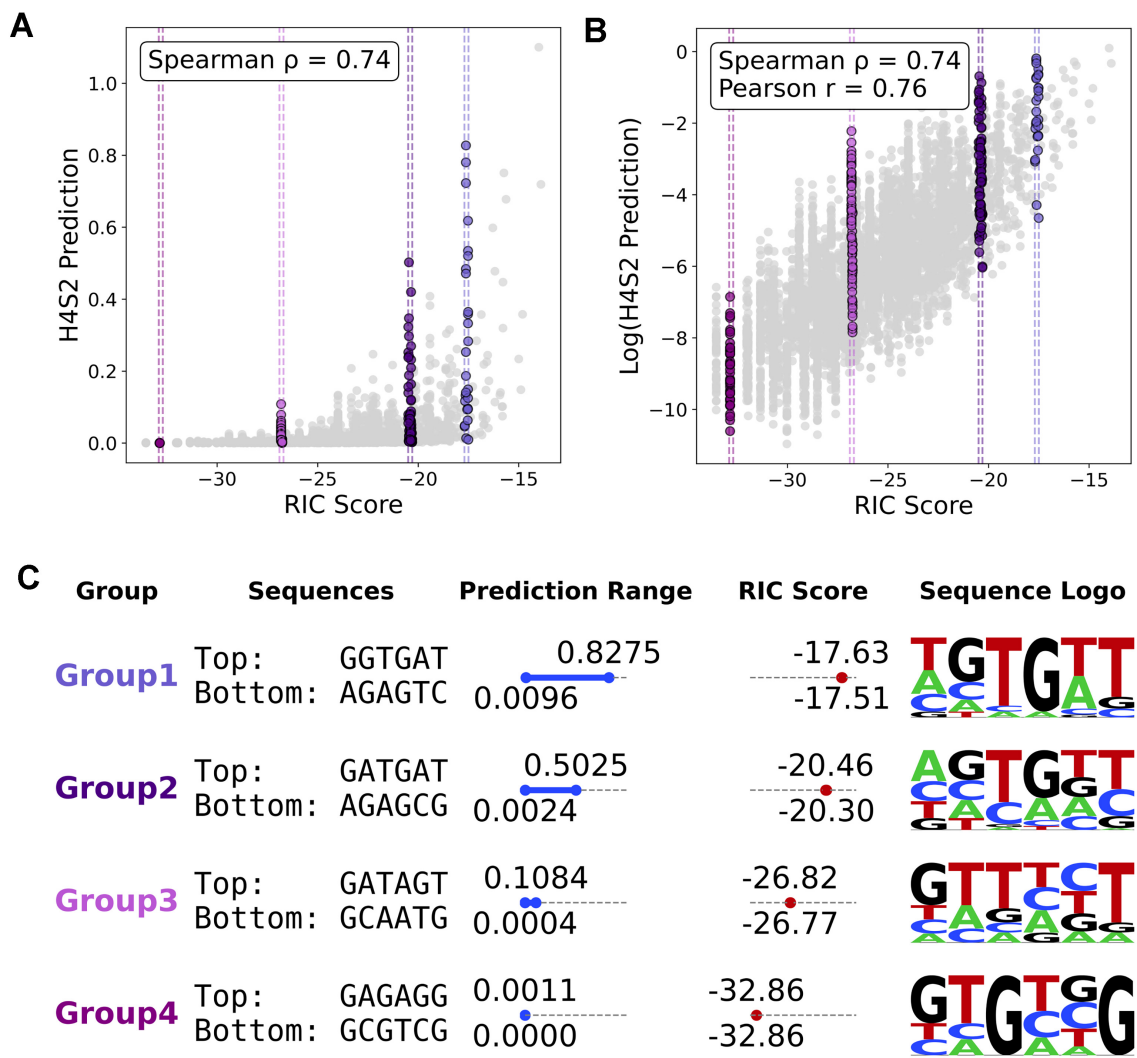
(**A**) A scatter plot of the H4S2 model prediction and the RIC score, with a Spearman rank order correlation of 0.74 *ρ*. (**B**) A scatter plot of the log transformed H4S2 model prediction and the RIC score, with a Spearman rank order correlation of 0.74 *ρ* and Pearson correlation, *r* = 0.76. Four vertical striations are highlighted that are sampled near RIC scores of ­−17 (Group1), ­−20 (Group2), ­−26 (Group3), and ­−32 (Group4). (**C**) A set of descriptive information on the sampled vertical striations, include the top and bottom H4-S2 sequences within the striation, the range of the H4S2 model prediction, the range of the RIC score, and the sequence logo for the striation.

The RIC score is a statistical model that can discriminate between functional and nonfunctional RSSs [42]. By using the correlation of mutual information between marginal and joint probability distributions of different arbitrary position combinations, Cowell *et al.* designed the RIC score algorithm based on the sequence conservation of mouse endogenous RSSs [42]. RSSsite is a webtool that calculates the RIC score for a given DNA sequence “www.itb.cnr.it/rss/help.html” [21]; however, we instead utilized a script that can be implemented within an interactive notebook. The RIC algorithm PERL script was created by Cowell *et al.* [42] but access is now deprecated. We recovered the script using Internet Archive’s Wayback Machine (https://web.archive.org/web/20120624013846/http://www.duke.edu/∼lgcowell/). By using the updated list of endogenous human RSSs found on the RSSsite, we ensured the PERL script’s RIC score calculation matched the RSSsite’s calculation (data not shown). The PERL script was used to compare RIC scores to the H4S2 model predictions as described in Results and shown in Fig. 3A–C. The RIC algorithm PERL script is available in supplementary data and repositories.

### Predicted effects of mutations in the H4-S2 region on recombination efficiency

To evaluate the effect that every theoretical DNA single point mutation within the H4-S2 region may have on V(D)J recombination efficiency, we compared the predicted recombination efficiencies of each nucleotide at each position in the H4-S2 region. First, we selected a set of sequences with a given nucleotide at a specific position, then for each possible mutation we selected the mutated set of sequences that contain the mutated nucleotide. As an example, for point mutation A→T at H4, we pulled all samples that matched the original nucleotide (H4-S2: “ANNNNN”) and all samples that matched the mutated nucleotide (H4-S2: “TNNNNN”). Second, we measured the difference between the two sets to quantify the average change in recombination efficiency. This was done for every point mutation at each position of the H4-S2 region. The effect of point mutations on the H4S2 model prediction and on the RIC score were compared by making separate heatmaps for each position (Supplementary Fig. S7).

### Heptamer melting propensity

**Figure 4.**
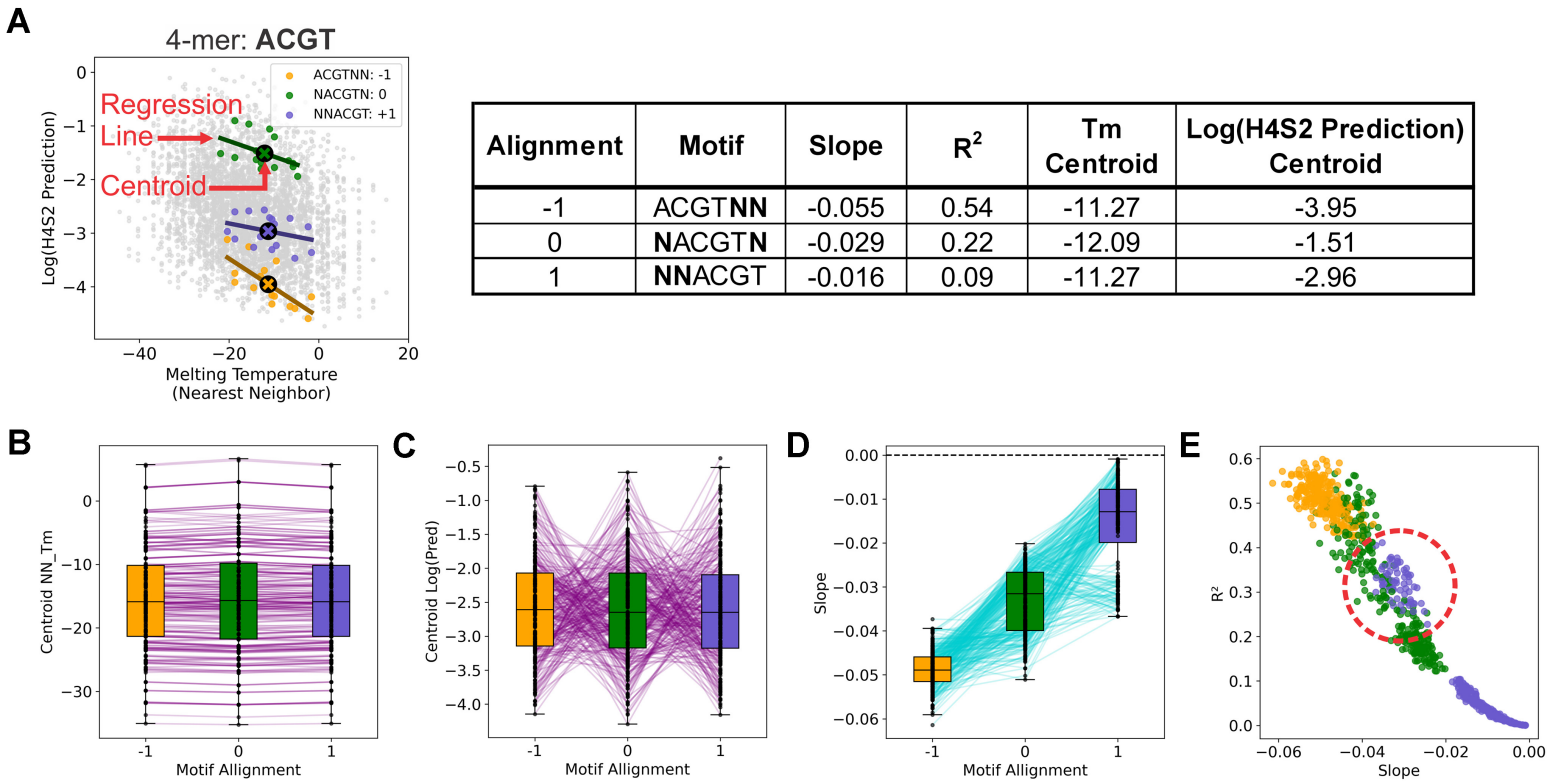
Analysis of the sequence alignment’s effect on melting temperature for all 4 nt long k-mers. All possible 4-mers were generated (*n* = 256). Then each 4-mer was positioned within the H4-S2 region to give three different alignments, referred to as alignment -1 (4-mer at H4-H7, colored yellow, ‘4-mer’NN), alignment 0 (4-mer at H5-S1, colored green, N’4-mer’N), and alignment + 1 (4-mer at H6-S2, colored lavender, NN’4-mer’). Independent linear regressions were performed on all three alignments for each 4-mer sequence, where the regression was conducted between the log transformed H4S2 model prediction and the melting temperature. (**A**) An example of *T*_m_ analysis for different alignment motifs is shown using the 4-mer ‘ACGT’. Sequences that match each alignment (as noted in the plot’s legend) were pulled. Each alignment set contains 16 different sequences. Linear regression was performed on each alignment set, and the slope, *R*^2^, and centroid for each set determined. The resulting values for the ACGT alignments and the log(H4S2 prediction) are listed in the table. (**B**) A box plot of the *T*_m_ centroid for the distribution of samples that match a given 4-mer sequence and alignment motif. (**C**) A box plot of the log transformed H4S2 model prediction centroid for the distribution of samples that match a given 4-mer sequence and alignment motif. (**D**) A box plot of the slope of each regression. Purple (B and C) and cyan (D) lines connect the same 4-mer sequence through the three different alignments (−1, 0, +1). Together, all 4-mer sequences, regardless of sequence content or alignment, showed a consistently negative relationship with melting temperature within the H4-S2 region (See Supplementary Dataset S2 for individual regression results). (**E**) A scatter plot of each regression line’s slope and *R*^2^ fit metric for all possible 4-mer sequences and their three alignments. The red dashed circle highlights the +1 alignment values with the higher *T*_m_ dependence.

The melting temperature of the H4-S2 region was calculated using Biopython library’s MeltingTemp module with the nearest neighbor method [43]. We performed a Spearman rank order correlation between the melting temperature and the H4S2 model prediction (Supplementary Fig. S8A and B). To explore the relationship between melting temperature in the context of sequence content and sequence alignment, we generated motifs with three different sequence alignments within the H4-S2 region and then performed linear regression between the melting temperature and the H4S2 model prediction. The analysis of the resulting regression metrics is shown in Fig. 4 and Supplementary Fig. S8C–F.

### SHapley Additive exPlanations—first-order interactions

The SHAP Python package was used to algorithmically estimate how each nucleotide-position feature increased or decreased the model’s prediction [44]. The package’s default explainer selected the permutation algorithm to calculate the SHAP values, where the sum of all the feature’s SHAP values for any given H4-S2 sequence in a 12-RSS and the average model prediction (base prediction) across all samples will equal the model’s prediction of the V(D)J recombination efficiency for that DNA sequence. SHAP summarizes a model’s learned complexities by marginalizing each feature’s many connections and interactions into one single contribution for every sample; this provides a powerful technique in interpreting complex models while also being capable of estimating the varying contribution of a feature based on the broader context of the other input features.

### Novel pairwise SHAP value comparative analysis—second-order interactions

Here, we developed a new method to perform a pairwise comparison of the SHAP values between two nucleotide-position features. Specifically, for each comparison of two binary features, we produced a scatter plot of SHAP values for each of the four combinations of possible binary values (Supplementary Fig. S9). For each combination of binary values, the SHAP values tended to fall along a half-line extending from the origin along some direction. We estimated the half-line's direction and maximum distance to profile how two features work together to influence the model’s prediction.

Each comparison resulted in a multimodal (or multipeak) distribution where the combination of two feature’s binary (0 = absence, 1 = presence) encodings (0,0; 0,1; 1,0; 1,1) coincided with the modes (or peaks) of the pairwise SHAP value distribution. The multimodal distributions were partitioned by the combination of two feature’s binary encoding, where each mode formed a vector-like distribution of distinct direction and length that originated from the origin. Principal component analysis (PCA) was used to quantify the direction and shape of each mode’s cooperative relationship vector (CRV). The eigenvector of PC1 was used to describe the slope of the CRV, and the distribution’s original centroid was used to describe the direction (positive or negative) of the CRV. To reduce the sensitivity of outliers, the length of the CRV was determined by Pythagorean theorem using the 99th percentile of the absolute values of both variables’ SHAP values as the X and Y components (Equation 1) as X_99_ and Y_99_, respectively. The angle of the CRV was calculated from the positive *x-*axis to the vector in a counterclockwise direction using the four-quadrant inverse tangent (Equation 2). The CRV quantifies the typical direction (angle) and the maximum strength (length) of how changes in one feature’s SHAP value affects another feature’s SHAP value (Supplementary Fig. S9). The angle describes the type of relationship (cooperative or antagonistic) that is observed, while length provides the near maximum strength of the observed interactions between two feature’s SHAP values across the different H4-S2 sequences.


(1) \begin{eqnarray*}
{\mathrm{Vector\;Length}} = \;\sqrt {\;{X_{99}}^2 + {Y_{99}}^2}
\end{eqnarray*}

(2) \begin{eqnarray*}
{\mathrm{Vector\;Angle\;}} = \;atan2\left( {{Y_{99}},\;{X_{99}}} \right)
\end{eqnarray*}

Each CRV was plotted on a scatter plot based on its length and angle, where each point is a description of how a model treats the binary encodings for two specific input features (Supplementary Fig. S10). Due to the number of CRVs being analyzed, the length and angle plots were simplified by grouping the CRVs by position (Supplementary Fig. S11). The length and angle plots were transformed by a cumulative summation function of the CRV lengths across each angle. The resulting curve marginalized over the angle metrics (*x*-axis) where the steepness of the line relates to the density of CRVs at a given angle and the height of the change relates to the strength of all the CRVs at a given angle.

As with the H4S2 model, the SHAP package was used to explain the H4S2-cNon model’s prediction for each feature. The SHAP values were evaluated and grouped by the corresponding nonamer sequence (CF1, Pax3, or LMO2). The mean absolute SHAP values for each feature was calculated as was done with the H4S2 model. Each of the CF1, Pax3, and LMO2 nonamer’s SHAP values were rescaled separately by dividing each value by each of the nonamer’s global mean or by that nonamer’s average mean absolute SHAP values, as shown in Fig. 8. This recentered the SHAP values, redefining each point as the fold change from that specific nonamer’s global mean (Supplementary Fig. S12A). The two-sample KS test compared each feature’s rescaled SHAP value distributions and if they are likely or unlikely to share a similar distribution (Supplementary Fig. S12B–G). Dividing by each nonamer’s global mean reduced and normalized the major differences in scaling between the SHAP value distributions allowing the KS test statistic to better focus on the underlying shape of each nonamer’s distributions. Candidate nonamer-specific long distance second-order interactions were identified in a series of two-way comparisons between the three nonamer dataset’s and their rescaled SHAP values.

### Fluorescence-based cellular V(D)J recombination assay

A schematic for RAG-mediated V(D)J recombination of pMAX-INV-derived plasmid substrates leading to green fluorescence protein (GFP) expression in the cellular assay is shown in Supplementary Fig. S2B. Plasmid substrates for the fluorescence-based V(D)J recombination assay were constructed from the parent plasmid pMAX-INV by replacing the 12-RSS, flanked by MluI and EcoRI sites. To replace the original 12-RSS in pMAX-INV, oligonucleotides (IDT DNA, Inc.) with complementary 12-RSS sequences were annealed resulting in MluI and EcoRI compatible overhangs on the 5′ and 3′ ends of the oligonucleotide duplex (Supplementary Table S1). The annealed oligonucleotide duplexes were phosphorylated using T4 DNA kinase and subsequently ligated using T4 DNA ligase into the parent pMAX-INV plasmid that had been previously linearized by digestion with MluI and EcoRI. As plasmid ligated with the oligonucleotide duplex insert lacked an MluI site, the ligation mixes were incubated with MluI (to reduce circularized parent plasmid levels) prior to transformation into *Escherichiacoli* DH5α cells. The correct DNA sequences of isolated plasmids were confirmed by whole plasmid sequencing (Plasmidsaurus, Inc.). The DNA sequence for the partner 23-RSS in each plasmid substrate is 5′-CACAGTGGTAGTACTCCACTGTCTGGCTGTACAAAAACC-3′. Enzymes and competent *E. coli* cells were from New England Biolabs.

The fluorescence based cellular V(D)J recombination assay was done as recently described [45] with modification as described below. For mammalian cell transfections, Expi293 cells (Gibco, Carlsbad, CA) were plated at 1 × 10^6^ cells/ml in six-well plates in Gibco Expi293 expression media on day one. On day two, cells were co-transfected with three separate vectors: a plasmid substrate and the mCherry-core RAG1 and mCherry-RAG2 expression vectors. Transfections were performed using a 4:1 reagent:DNA ratio using ExpiFectamine 293 transfection reagent (Gibco). The fluorescence signal from the mCherry tag was used to assess transfection efficiency. The Cytek Aurora flow cytometer (Cytek Biosciences, Fremont, CA) at the Oklahoma Medical Research Foundation flow cytometry facility was used to measure GFP expression 48 h post-transfection. Live cells were gated based on mCherry positive cells, and GFP positive cells were quantified from the live, mCherry positive cell population. Flow cytometry data for each condition were normalized to the positive control (RSS-1) to determine the percent of GFP positive cells for *n* = 3 experiments. Negative control conditions for recombination activity of the RSS-1 plasmid substrate was performed in parallel and included transfection in the absence of the mCherry-core RAG1 vector. Statistical tests were performed in GraphPad Prism 9 using an ordinary one-way ANOVA with Dunnett’s multiple comparisons test. As stated in the appropriate figure legends, asterisks denote *P*-value range, where *P < 0.05; **P < 0.01; ***P < 0.001, and ****P < 0.0001.

### Use of generative AI in script development

The free and available OpenAI’s ChatGPT and Google’s Gemini models were used from 2022 to 2025 in the development and debugging of Python scripts for the use in Jupyter Notebooks. The Python scripts were used to process data, build neural network models, employ SHAP model explainability techniques, perform CRV analysis, and create plots. Working in code blocks, the researchers directed and guided all code generation.

We inspected and ran each AI generated version of code, then we re-prompted the AI model with relevant information to improve the current code block. We provided the context of the current or dependent code blocks; we also provided the code’s outputs and any errors. Critically, we identified flaws in logic and provided feedback to improve the code block. Through iterative improvements, errors in the code logic were resolved and tested. The final Python scripts and notebooks are available through a Zenodo Repository (https://doi.org/10.5281/zenodo.14903647) and a GitHub Repository (https://github.com/A-Blue-Jay/Building-a-Neural-Network-Models-Notebooks).

## Results

### Model input dataset—SARP-seq N(H4-S2)

To generate a neural network model for RSS selectivity, sufficient experimental data on V(D)J recombination activity is required (Fig. 1). To this end, we utilized previously-generated results obtained with the SARP-seq method [29] (Supplementary Fig. S2A). The experimental results from the plasmid-based SARP-seq approach represents an unbiased analysis of RSS selectivity that is primarily based on differences in DNA sequence specificity to the 12-RSSs (Fig. 1C). The recombination activity varied widely for the 12-RSSs in the plasmid library, resulting in an extremely asymmetric distribution (Supplementary Fig. S3C). Overall, the complete SARP-seq iSeq dataset (consisting of 4096 sequences in total) showed, to the nearest percent, 35% 12-RSSs with read counts equal to or above 10 reads, 21% 12-RSSs with read counts below 10 reads, and 43% with no recombination at 0 read counts (Fig. 1D).

### Overview of generating the H4S2 model

As described in “Materials and methods” section, a dense neural network model was trained using the 12-RSS DNA sequence at positions H4-S2 as feature inputs and a min-max normalized read count as the target prediction (Fig. 2A). To train the H4S2 model, the SARP-seq dataset first required preprocessing, followed by optimization of the model’s hyperparameters (Supplementary Figs S3 and S4). Final training and testing of the H4S2 model, as described in “Materials and methods” section, resulted in a robust model that effectively predicts the V(D)J recombination efficiency level for different 12-RSS sequences (Fig. 2), albeit with the sequence coverage limited to positions H4-S2. The remaining spacer (S3-S12) and nonamer (N1-N9) were not included in the model inputs as these sequences were constant in the SARP-seq experimental design. While data are currently limited, regions such as the nonamer and the coding flank, (the sequence 5′ of the RSS sequence) have shown little effect on the H4-S2 region’s sequence selectivity on recombination activity [29]. The H4S2 model by extension provides a detailed representation of the effect the H4-S2 region's DNA selectivity has on V(D)J recombination levels.

In a pre-processing step, as described in “Materials and methods” section, the 12-RSS sequence H4-S2 positions were converted using one-hot encoding, where each nucleotide base for every position of the sequence is represented by its own binary feature. For example, if position H4_A is an A nucleotide, then H4_A’s binary feature value would be one, while the H4_T, H4_G, and H4_C’s binary feature value would be zero (Supplementary Fig. S1A). Using each binary nucleotide-position feature, the H4S2 model was trained to predict the level of RAG-mediated V(D)J recombination—essentially how likely a 12-RSS is to undergo V(D)J recombination. By employing stratified *k*-fold cross-validation, we determined that a simple architecture containing two hidden layers with early-stopping and dropout regularization yielded the best performance with respect to each rotation’s validation dataset (Supplementary Fig. S3A and B).

We used the mean squared error (MSE) and FVAF of the training and validation subsets to select the optimal H4S2 model's hyperparameters and architecture (Supplementary Fig. S3F). After cross-validation optimization, a final exploration of the hyperparameters was performed to ensure that the model configuration was optimized using the above performance metrics (Supplementary Fig. S4).

After optimization, the trained H4S2 model showed little sign of overfitting to the training (0.85 FVAF) and validation (0.84 FVAF) subsets. The lower FVAF performance seen when predicting the test subset (0.74 FVAF) (Fig. 2B) is lower than the training and validation subsets’ performance but is within the range observed during *k*-fold cross-validation. The trained H4S2 model was then tested against previously unseen SARP-seq data, as described in “Materials and methods” section (Fig. 2C and D). Both FVAF (0.90 FVAF) and Spearman correlation (∼0.75 *ρ*) metrics showed that the model was able to generalize the unseen SARP-seq data (Fig. 2E and F), and it does so with low error (∼0.02 RMSE) (Fig. 2G).

The final H4S2 model’s residual error of the training replicate had only a few sequences with errors larger than ±0.2 prediction units, where 1 represents the highest observed recombination level, and 0 is zero recombination or is unlikely to recombine. The correlation between the residual error to the standard deviation across the training, iSeq1, replicate and two testing, iSeq2 and miSeq1, experimental replicates had a Spearman correlation that was slightly negative (−0.12 *ρ*) suggesting sequences have a slight skew toward underprediction (Supplementary Fig. S5A). Although slightly skewed negatively, most of the points have nearly zero residual error (Supplementary Fig. S5B). A strong Spearman correlation (0.78 *ρ*) was observed between the standard deviation of experimental replicates and the absolute residual error of the training dataset, indicating a strong positive relationship between the errors of the model and the SARP-seq experiment (Supplementary Fig. S5C). Thus, the model’s performance is reliant on the quality of the experimental data. Both the absolute residual error of the training dataset and standard deviation across experimental replicates had an asymmetric distribution that both skewed toward zero (Supplementary Fig. S5D and E) indicating that a large majority of the sequences have little to no residual error and little to no variability between experimental replicates. One exception is the H4-S2 sequence AGTG|CT, which is evident by the prediction’s small absolute residual error to the training dataset while having a large standard deviation in measured recombination efficiency across replicates for that particular sequence (Supplementary Fig. S5A and C). Together, this demonstrates that the model has accurately built and trained a mathematical model of the SARP-seq N(H4-S2) experiment, and that it is able to generalize across experimental replicates, with few discrepancies.

### H4S2 model performance compared to the historic RIC score metric

The RIC score is a metric developed over 20 years ago to discriminate RSS functionality [42]. While it generally can discriminate between active and inactive RSS substrates, it struggles to determine the relative level of recombination activity for active RSSs [19, 20, 42, 46, 47]. The RIC score utilizes the sequence-based conservation of the endogenously known RSSs to generate a negative value that can effectively discriminate between functional and nonfunctional RSSs, where −38.81 to 0 is the established threshold range for functional 12-RSSs and negative infinity to −38.81 are labeled as nonfunctional [42]. A comparison between the RIC score and the H4S2 model’s prediction of recombination efficiency for the entire SARP-seq N(H4-S2) dataset yielded a positive correlation (Spearman correlation of 0.74 *ρ*) (Fig. 3A), where a log transformation of the H4S2 model’s prediction eliminated the nonlinear aspect of the correlation (Pearson correlation of *r* = 0.76) (Fig. 3B). Notably, vertical striations are evident throughout the correlated distribution, which correspond to 12-RSS H4-S2 sequences that the RIC score does not distinguish (resulting in similar scores), while the H4S2 model results in predictions of vastly different recombination frequencies for the same set of sequences. The sampled vertical striations at RIC score ­−17, ­−20, ­−26, and ­−32 showed that the prediction range increased in variability as the RIC score increased toward zero (Fig. 3C). Across the sampled vertical striations, the top and bottom predicted sequences had little sequence similarity. Each vertical striation had a diverse sequence logo, especially at positions H4 and S1 (Fig. 3C).

Next, we determined the effect that DNA point mutations within the H4-S2 region had on the model prediction. At each position within the H4-S2 region, the average change in the H4S2 model prediction was determined for each possible point mutation. Point mutations of the canonical G, T, or G at positions H5, H6, and H7, respectively, resulted in a negative change in the recombination efficiency prediction. Further, point mutants from C to any other base at position H4 has a positive change to the H4S2 model’s prediction and mutating from T to any other base at S2 has a negative change to the H4S2 model’s prediction. Further, the effect of the DNA point mutations on the RIC score further revealed that the two recombination metrics had different profiles on how point mutants effect recombination in the H4-S2 region (Supplementary Fig. S7A and B).

### Melting propensity of the H4-S2 region

RAG-mediated DNA cleavage requires substantial conformational changes of the RSS, such as base unpairing. As the energetics of DNA base unpairing is reflected by the melting temperature (*T*_m_), we investigated melting temperature’s association with RAG mediated recombination. The *T*_m_ (NN_Tm) was calculated for each of the 4096 H4-S2 12-RSS substrates by using the nearest neighbor model’s prediction of melting temperature (as described in “Materials and methods” section). The *T*_m_ (NN_Tm) not only considers the hydrogen bonding between base pairs but also the base stacking energies of neighboring nucleotides [48]. The log transformed H4S2 model prediction versus the calculated NN_Tm values for each DNA sequence were weakly and negatively correlated, with a Spearman correlation of −0.28 *ρ* and Pearson correlation of *r*= −0.29 (Supplementary Fig. S8A and B). A decreased melting temperature indicates a DNA molecule is intrinsically less stable and by extension would have more dynamic movement. This decrease in DNA stability generally coincides with improved recombination efficiency levels.

As a sequence’s melting temperature depends highly on sequence content, we designed a motif alignment scheme that minimized disturbances in the neighboring base stacking energies and in the G/C content. This allowed us to explore the effect of the motif's sequence content, as well as the effect of sequence alignment, on recombination activity. To perform an extensive analysis, we generated all possible 4-mer sequences with A, T, G, and C bases (*n* = 256 4-mer sequences). Each 4-mer sequence was shifted within the H4-S2 region for the following alignments: -1, (4-mer)NN; 0, N(4-mer)N; +1, NN(4-mer). We then pulled sequences with identical 4-mer sequences (i.e. -1, ACGTNN; 0, NACGTN; +1, NNACGT) for each of the 256 4-mer sequences and performed a linear regression between the melting temperature and log transformed H4S2 prediction for each of the three alignments where slope, fit metrics, and centroid were determined for each regression (see example in Fig. 4A). The regression results of all possible 4-mer sequences and each sequence alignment is listed in Supplementary Dataset S2. Plotting the centroids versus *T*_m_, showed that the *T*_m_ did not substantially change across the three different alignments for each 4-mer (Fig. 4B). However, the alignment motifs of the different 4-mer sequences had various impacts on the H4S2 prediction (Fig. 4C). As exemplified in Supplementary Fig. S8C–E, motifs that contain alternating R,Y sequences showed the largest changes in the model predictions for recombination activity between the −1, 0, and + 1 alignments, while a R-rich motif had no dependence on alignment (Supplementary Fig. S8F). These results are consistent with the preference for an RYR motif at positions H5-H7 [29].

For our *k*-mer melting analysis, every linear regression line had a negative slope, across all 4-mers at all three alignments (Fig. 4D). In essence, each individual linear regression measured the effect that the two degenerate dinucleotides of a given alignment motif had on the relationship between melting temperature and the model prediction for a given 4-mer sequence. Regardless of the H4-H7 4-mer sequence content, the −1 sequence alignment motifs (motif at H4-H7 such as ‘4-mer’NN) showed the best fit regression lines with the highest *R*^2^ fit metric and with the most negative slopes (Fig. 4D and E, colored in yellow). As each regression is conditional on a given alignment motif’s sequence content and alignment, the slope demonstrates how sensitive the two degenerate nucleotide positions of an alignment motif are to melting temperature. Thus, the melting dynamics of the base pairs present at the S1-S2 dinucleotide can have a substantial effect on the recombination activity of any particular heptamer sequence. In contrast to the −1 alignment, the +1 sequence alignment motifs (4-mer at H6-S2, NN at H4-H5) had the smallest negative slopes (Fig. 4D and E). However, the regression analysis of the +1 motifs in Fig. 4E were clearly bifurcated into two subsets. The lower subset represents 4-mers that are insensitive to the Tm properties of the H4-H5 dinucleotide, while the upper subset (circled in Fig. 4E) shows increased *T*_m_ dependence on the H4-H5 dinucleotide. Notably, every 4-mer that contains a G at position H6 is located in the upper subset. Thus, unlike the S1-S2 dinucleotide, the Tm dependence of H4-H5 dinucleotide is selective for only certain sequence compositions in the 4-mers. We have previously reported that G is the least preferred base at position H6. Here, we demonstrate that the disfavored base at this position may be partially compensated by the melting dynamics of the adjacent H4-H5 dinucleotide. Overall, this analysis method will be important in future studies to determine the relative contributions of sequence content, alignment, and biophysical properties for the entire DNA recognition site.

### H4S2 model SHapley Additive exPlanations (SHAP) reveal nucleotide:position first-order interactions

**Figure 5.**
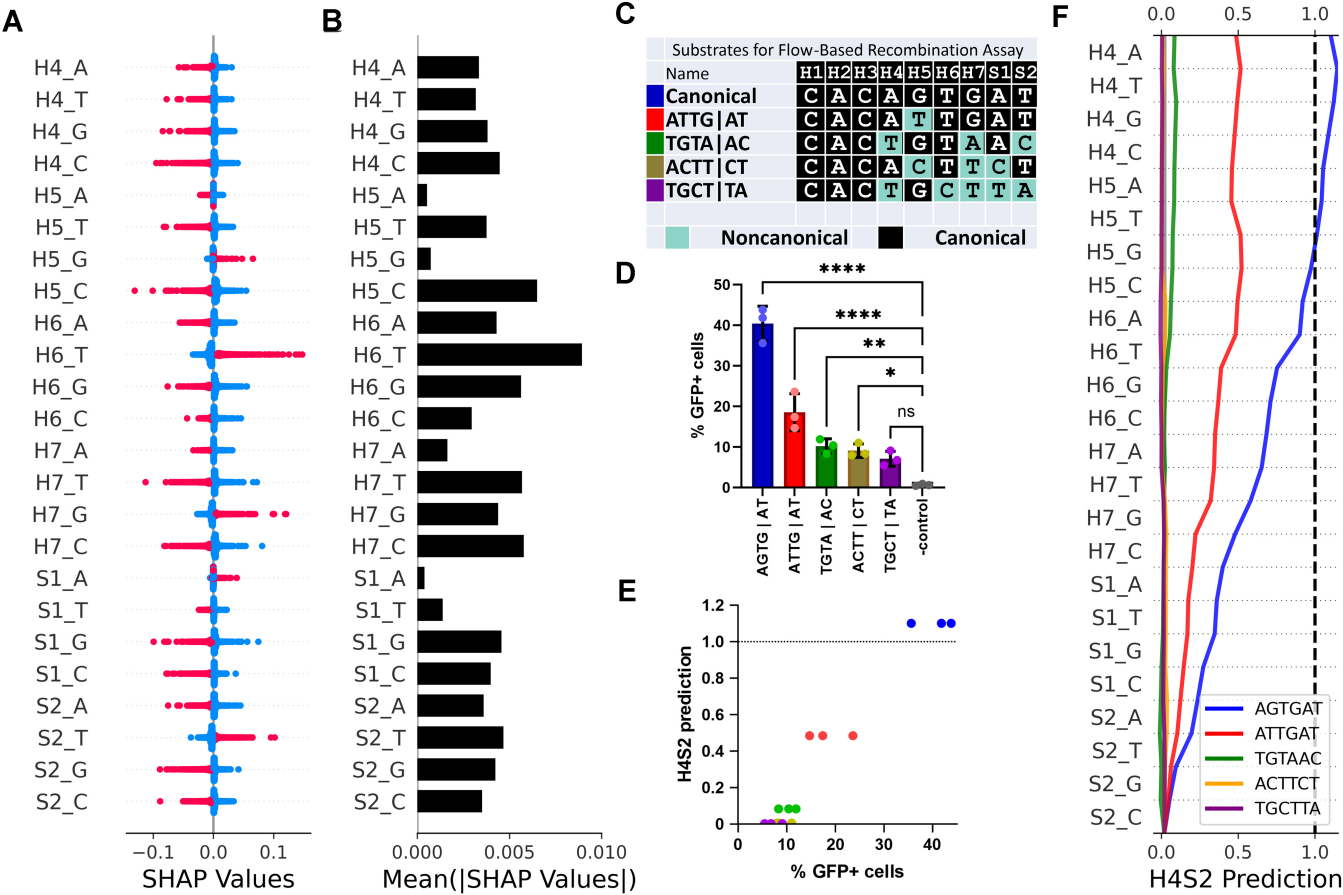
(**A**) The contribution of each feature’s SHAP value for every 12-RSS in the complete experimental iSeq1 dataset (Supplementary Dataset S1) to the H4S2 model’s prediction. Nucleotides present in the 12-RSS sequence at a particular position are shown as red symbols, while nucleotides absent in the sequence are represented as blue symbols. (**B**) The mean of the absolute value of the SHAP values, representing feature importance and total contribution or nondirectional change to the H4S2 model’s prediction. (**C**) The sequence of the 12-RSS heptamer and two adjoining spacer positions that were used in the plasmid substrates in the fluorescence-based V(D)J recombination assay, where (black) identifies sequence identity with the canonical sequence, and (teal) indicates nucleotides which varied from the canonical 12-RSS (shown in Fig. 1B). (**D**) V(D)J recombination on the plasmid substrates containing the indicated 12-RSSs and measured by the fluorescence-based V(D)J recombination assays. V(D)J recombination activity is plotted as %GFP-positive cells for each 12-RSS-containing plasmid. The sequences shown on the horizontal axis are the H4-S2 sequences in each 12-RSS. The negative control used the AGTG|AT plasmid substrate in the absence of a RAG1 expression vector in the recombination assay. Pearson *r*= 0.97 (**E**) Relationship between H4S2 model prediction and the independently measured recombination activity of the five separate 12-RSSs. (**F**) Cumulative SHAP values corresponding to the H4S2 model’s prediction for independently measured 12-RSSs. The asterisks denote the P-value range for an ordinary one-way ANOVA with Dunnett’s multiple comparisons test, where ns is not significant; *P < 0.05; **P < 0.01; and ****P < 0.0001.

To explain individual predictions made by the H4S2 model, we turned to a game theoretic approach known as Shapley Additive exPlanations (SHAP). SHAP is a model agnostic approach that is compatible with many model types to explain the contributions of feature variables on a model’s output, where positive SHAP values indicate an increase to the model’s prediction and negative SHAP values indicate a decrease to the model’s prediction [44, 49]. Here, SHAP was used to calculate the contribution of each feature nucleotide (A, T, G, C) at every position (H4-S2), as described in “Materials and methods” section. The resulting contributions explain how each feature is treated by the model. The distribution of SHAP values for each 12-RSS in the training SARP-seq N(H4-S2) dataset is bimodal. The modes for the bimodal distributions can be described by whether or not that nucleotide at that position was present or absent in that 12-RSS sequence (Fig. 5A). For the majority of features, the bimodal distribution’s modes show a clear separation between positive and negative SHAP values, with the distinct boundary near zero.

According to the SHAP first-order interactions, H4 had no preferred nucleotides that increased the model's prediction of the level of recombination efficiency. All other positions H5, H6, H7, S1, and S2 had one specific nucleotide whose presence consistently increased the model's prediction, while the presence of the other three nucleotides decreased the model’s prediction at those positions. Together, the SHAP values provide quantification of the first-order interactions that each nucleotide:position feature has on V(D)J recombination efficiencies.

The presence of nucleotides T at H6, G at H7, and T at S2 have strong positive contributions to the model’s prediction compared to H5_G and S1_A (Fig. 5A). In considering absolute contributions that any feature has on the model, H6 and H7 positions had the highest mean absolute SHAP values, indicating that these are the most impactful positions to the H4S2 model prediction (Fig. 5B). In particular, H6_T had the largest range of SHAP values, such that the model’s prediction heavily relies on this position (Fig. 5B). This indicates a critical function of the T nucleotide at position 6 in the 12-RSS heptamer, and is consistent with high-resolution structures showing one of the few sequence-specific interactions between RAG1 and the H4-S2 region of the RSS heptamer [24].

Interestingly, nucleotide H5_A and H5_G have small mean absolute SHAP values while H5_T and H5_C have higher mean absolute SHAP values. A similar dichotomy is seen at position S1, where S1_G and S1_C have higher mean absolute SHAP values while S1_A and S1_T have lower mean absolute SHAP values (Fig. 5B). While these groups of features have similar levels of mean absolute SHAP values, their SHAP value distributions do not look similar (Fig. 5A).

### The H4S2 model and experimentally determined recombination efficiencies are strongly correlated

To test V(D)J recombination activity experimentally, we optimized a cellular V(D)J recombination assay in which the activity of a 12-RSS, paired with a canonical 23-RSS on a plasmid substrate, was measured using flow cytometry (Supplementary Fig. S2B). Here, the V(D)J recombination activity on five plasmid substrates, each containing a different 12-RSS, was determined (Fig. 5C). The tested RSS substrates showed a diverse range of levels of V(D)J recombination efficiencies by an independent flow-based recombination assay (Fig. 5D). The H4S2 model effectively captures the diverse range obtained from the fluorescence-based assay, with a tight relationship between the model’s prediction and the experimental results for each 12-RSS (Fig. 5E). Decision plots provide a direct visualization as to how the sum of the features of each 12-RSS’s SHAP values yields the model’s prediction for that 12-RSS, providing useful insight into the nucleotides that provide the largest positive or negative contributions to the predicted recombination activity (Fig. 5F).

### A novel pairwise comparison of SHAP values of the H4S2 model illustrates second-order interactions

**Figure 6.**
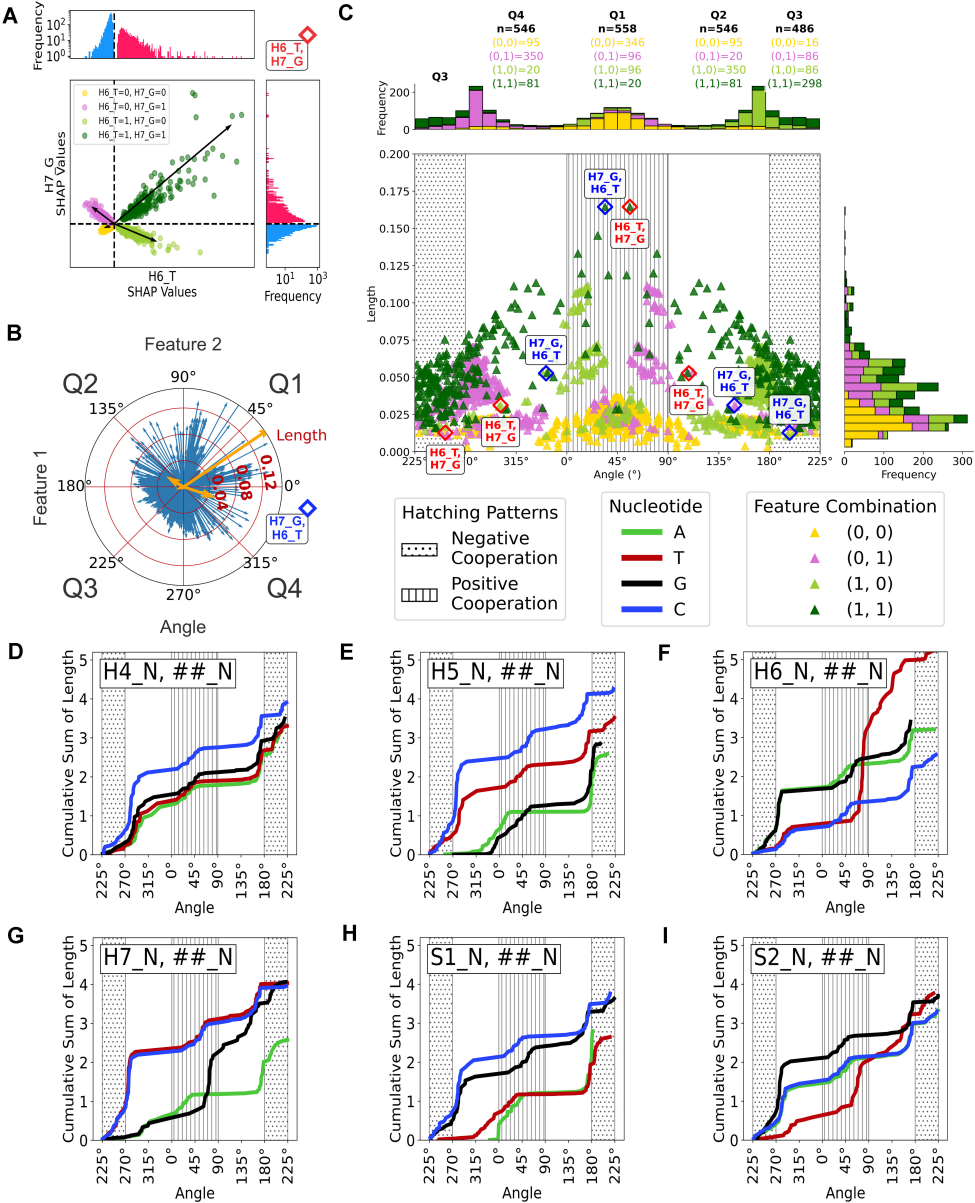
(**A**) A Multimodal pairwise comparison of two features’ SHAP values (Feature 1: position H6_T and Feature 2: position H7_G). The modes are explained by the combination of binary features encodings where green is 1,1; light green is 1,0; pink is 0,1; and yellow is 0,0. Each black arrow is a CRV fit by PCA and characterizes a single modality of the multimodal joint distribution. Histogram plots along each axis displays each feature’s SHAP values. (**B**) Polar plot projection of all cooperative relationship vectors between every combination of two features and their four combination of binary feature encodings (0,0; 0,1; 1,0; and 1,1) (blue). Vectors from Feature 1: position H7_G and Feature 2: position H6_T comparison are colored in yellow. (**C**) The length and angle plot of all CRVs between each feature and every other feature. CRVs are colored by their feature combination where green is 1,1; light green is 1,0; pink is 0,1; and yellow is 0,0. The histograms along each axis displays the distribution of CRV’s length and the distribution of CRV’s angle. The table above the plot quantifies the number of CRVs present in each quadrant by each feature combination. The points with red outline are CRVs that correspond with Feature 1: position H6_T and Feature 2: position H7_G. With reverse ordering and a blue outline are CRVs that correspond with Feature 1: position H7_G and Feature 2: position H6_T. (**D**­**–I**) grouping CRVs by their shared feature 1 (e.g. H4_A, ##_N). The cumulative summation of the vector length as the vector angle sweeps from 225° counterclockwise around the circle, for the feature 1 and all its pairwise comparisons. The cumulative summation distribution for (D) position H4, (E) position H5, (F) position H6, (G) position H7, (H) position S1, and (I) position S2 and the 4 features at each position derived from one-hot encoding. Supplementary Figure S11 shows the vector length and angle plots for each position and its 4 nucleotide features prior to cumulative summation. (D­–I) Plot regions with vertical line hatching patterns highlights Q1 and CRV’s with positive cooperation, and regions with dotted hatching patterns indicate Q3 and CRVs with negative cooperation.

SHAP values provide first-order contribution information on how a nucleotide-position impacts a model’s prediction but lacks second-order interaction information on how two nucleotide position features interact to impact a model’s prediction. Unfortunately, the permutation explainer used to algorithmically estimate SHAP values does not support SHAP interaction values that explore higher order interactions. Alternatively, dependency plots are typically used. However, as our encoded input data is categorical instead of continuous, the dependency plots provide limited information about higher order interactions. To account for these deficiencies, we developed a novel analysis of the SHAP values where each nucleotide position feature’s SHAP value is compared to every other nucleotide position feature’s SHAP value (Supplementary Fig. S9). As an example, the pairwise distribution between H6_T and H7_G demonstrates how the bimodal SHAP values can be compared resulting in a multimodal distribution. Notably, each mode coincides with the combination of a features’ four possible combinations of binary encodings (0,0; 0,1; 1,0; and 1,1) (Fig. 6A). Each mode’s distribution originates at or near the origin and extends outward in any direction creating tight, vector-like distributions. We used PCA to fit a scaled vector to describe each mode or vector-like distributions (Fig. 6A and Supplementary Fig. S9). The vector’s length was calculated by the distance from the vector tip to the origin. The length corresponds to the maximum strength of the cooperative relationship between two features and their combinations of binary encodings. The vector’s direction was calculated as the counter-clockwise angle from the positive *x-*axis to the vector and defines the effect the CRVs have on the model (Table 1). Positive cooperativity corresponds to CRV angles that approach 45° (Quadrant 1), where both features have positive SHAP values and both features increase the model’s prediction. Negative cooperativity corresponds to CRV angles that approach 225° (Quadrant 3), where both feature’s SHAP values are negative and the model shows a negative response to both features. In summary, vector angles near 45° and 225° depict where both features’ SHAP values contribute equally (positively or negatively, respectively) to the model’s prediction. CRVs with angles approaching 135° (Quadrant 2), or 315° (Quadrant 4), have SHAP values with opposing signs indicating antagonism between the two features. In an antagonistic relationship one feature is dampening the contribution of another feature on the model’s prediction. CRV around 135° have negative sensitivity to feature 1 while CRVs ∼315° have negative sensitivity to feature 2. CRV’s located near the *x-* or *y-*axis: 0°, 90°, 180°, and 270° indicate one feature’s SHAP values are much smaller than the other feature’s SHAP values, while the feature with the largest SHAP values predominates the pairwise CRV interaction. The model is less sensitive to feature 2 for CRVs ∼0° and 180° than feature 1, while for the CRVs with angles near 90° and 270° are less sensitive to feature 1 than feature 2. Together, these multimodal distributions contain information about the second-order interactions between nucleotide:position features, where CRVs quantify how the contributions of one feature changes in relation to the contributions of another feature (Table 1).

**Table 1.** Interpretation of CRVs to analyze second-order interactions

**Interpretation of CRVs by quadrant**			
**Quadrant**	**Angles**		**Description of pairwise interactions**
**Q1**	0°	90°	Model has a POSITIVE response to BOTH Features
**Q2**	90°	180°	Model only has a NEGATIVE response to Feature 1
**Q3**	180°	270°	Model has a NEGATIVE response to BOTH Features
**Q4**	270°	0°	Model only has a NEGATIVE response to Feature 2
**Interpretation of CRVs near an axis**			
**Axis**	**Angles**		**Description of pairwise interactions**
**Horizontal**	0°	180°	Feature 2 has smaller SHAP values than Feature 1
**Vertical**	90°	270°	Feature 1 has smaller SHAP values than Feature 2
**Interpretation of CRVs near a diagonal**			
**Half-line**		**Angles**	**Description of pairwise interactions**
***y* = *x*; when *x* > 0**		45°	BOTH features contribute EQUALLY but with SIMILAR signs
***y* = *x*; when *x* < 0**		225°	BOTH features contribute EQUALLY but with OPPOSING signs
***y* = −*x*; when *x* > 0**		315°	BOTH features contribute EQUALLY but with SIMILAR signs
***y* = −*x*; when *x* < 0**		135°	BOTH features contribute EQUALLY but with OPPOSING signs

A detailed description of the meaning of different CRV angle values. Explaining how two features interact to impact the model’s prediction.

For example, Fig. 6A depicts a pairwise comparison of the H6_T feature’s SHAP values (top histogram) and H7_G feature’s SHAP values (right histogram). The histograms are colored by the binary feature values, if the binary feature was present (red) or absent (blue). The scatter plot in Fig. 6A illustrates the multimodal distribution of pairwise comparison of the two features’ SHAP values, as colored by the combination of the two binary feature values of the features (0,0; 0,1; 1,0; and 1,1). With this representation, we can visualize how the H6_T and H7_G features interact together to influence the H4S2 model’s prediction. When both features are present (position H6 is nucleotide T and position H7 is nucleotide G), the points are colored dark green. The distribution itself and its CRV vector are in quadrant one, meaning there is positive cooperation between these SHAP values, and both features work together to contribute positively to the model’s prediction. Likewise, when both features are absent, the position H6 is either nucleotide A, G, or C while position H7 could be either nucleotide A, T, or C, and the points are colored yellow. This latter distribution and its CRV vector are located in quadrant 3 and correspond to a negative cooperative relationship, where both feature’s work together to decrease the model’s prediction. In this case, the yellow CRV suggests a small pairwise influence to the model’s prediction, while the dark green CRV, when both features are present, suggests there is a much larger influence on the model’s prediction. The pink and light green CRVs represent when one feature is absent and other is present. The CRVs for the H6_T and H7_G pairwise comparison are in quadrant 2 and 4, indicating they have antagonistic relationships. In effect, both features work against each other, where one feature increases the model’s prediction, while the other feature decreases the model prediction. The pink and light green CRVs are closer to the *x* axis and suggest the H6_T has larger contributions to the model’s prediction than H7_G, which agrees with the mean absolute SHAP values (Fig. 5B). Alone, each scatter plot can provide an tremendous amount of information on how the H4S2 model treats two features. Together, the collection of scatter plots from the pairwise comparison of each nucleotide:position features (Supplementary Fig. S9) provides a granular explanation of how the H4S2 model predicts V(D)J recombination efficiencies from DNA sequence while also capturing the second-order interactions between nucleotide:positions within the heptamer and adjoining spacer (Supplementary Fig. S10).

Overlaying all the cooperative vectors reveals many CRVs across all angles (Fig. 6B and Supplementary Fig. S10), with the majority of the 1,1 (dark green) CRVs being located in quadrant 3, which indicates that most pairwise interactions where both nucleotides are present, have negative cooperativity (Fig. 6C). While the CRVs that show positive cooperativity had a larger range of vector lengths, these are fewer in number, indicating that the model relies on many weaker pairwise-interactions to make its prediction. Across the different CRVs, the absent, absent binary-encoding features (0,0; yellow) were typically of smaller lengths, regardless of quadrant (Fig. 6C). It is also noteworthy that when a CRV’s feature 1 and feature 2 are paired in the opposite order the vectors are mirrored across a 225° and 45° diagonal line. For example, the opposite feature order of H6_T and H7_G is H7_G and H6_T, where their CRVs are mirrored and share the same length values (Fig. 6C, outlined points). Since the opposite order of the feature combination of 0,1 (pink) is reversed as 1,0 (light green), the reflected distribution is seen between the two groups across the 45° line (Fig. 6C).

### Cooperative relationship vector’s cumulative length and angle plots—explaining H4S2 model behavior

The vector length and angle plots summarize the strength and behavior of each pairwise comparison and their unique second-order interaction for each combination of the two feature’s binary encodings (Supplementary Fig. S10). CRVs that share a common feature typically cluster together. The vector length and angle plots for all CRVs visually demonstrate complex structure and patterns in how pairwise features work together to influence the H4S2 model’s prediction (Supplementary Fig. S10).

We simplified the vector length and angle plot by grouping CRV’s that include the features that share the same feature 1 and only displaying groups from the same position (Supplementary Fig. S11). The graphs capture how one feature interacts pairwise with all other features. For instance, some nucleotides shared similar CRV length and angle distributions with another nucleotide at the same position. For example, all the H4_A’s CRVs and all the H4_T’s CRVs covary and overlap at all angles (Supplementary Fig. S11A). The position-wise covariation between different nucleotides suggests these nucleotide-position features share similar second-order interactions and the model does not greatly differentiate them. The covariation between H4_A and H4_T and their feature’s second-order interactions indicate that the two nucleotide:position features do contribute to the model’s prediction by interacting with all other features in the same way. Conversely, partial covariation is seen in all the H4_G CRVs and all the H4_C CRVs because they only covary between 0° and 180° but not the other angles. This suggests some of H4_G’s pairwise interactions to other features are similar to H4_C’s pairwise interactions, but they are not all similar to H4_C’s CRVs (Supplementary Fig. S11A).

To make visual interpretation easier, the position-wise vector length and angle plots (Supplementary Fig. S11) were further transformed by cumulative summation (running total) of the vector lengths across the angles of each nucleotide (Fig. 6D–I). The change in slope of the cumulative length changes in relation to the density of CRVs in the length and angle plot for a given region (Supplementary Fig. S11), where a steep slope at 45° would indicate a high concentration of positive cooperative CRVs for that particular feature and its pairwise comparisons. The cumulative length and angle plots show covariation between features when the distributions share a similar shape, by confounding the density of the CRVs and their length at a particular angle.

The CRVs for the nucleotide-position features at position H4 showed strong covariation between H4_A and H4_T. The cumulative length and angle plot shows that H4_C has more CRVs in the negative cooperative regions of the plot (Fig. 6D), highlighting the model’s disfavor for H4_C. The shared shape between A, T, and G nucleotides at position H4 in the positive cooperative region indicates the model provides little differentiation between these three nucleotides at position H4. While the previous interpretations based on SHAP first-order interactions (discussed above) were that the H4 position had little nucleotide preference, our analysis based on the CRVs refines this perspective where the model slightly disfavors H4_C over the other nucleotides at position H4 (Fig. 6D).

Position H5’s CRVs revealed covariation between H5_T and H5_C, where both had many CRVs with negative cooperation and both had many CRVs that demonstrate a negative response to feature 2 (Fig. 6E). While the models had similar second-order pairwise interactions between H5_T and H5_C, H5_T had a steeper slope within quadrant 3 (Q3), which is indicative of either a dense population of negative cooperative CRVs or a group of CRVs with stronger negative cooperative relationships than what was seen with H5_C’s CRVs. This further indicates that the model had stronger disfavor for H5_C. Additionally, H5_A and H5_G also demonstrated covariation (Fig. 6E). Nucleotide:Position H5_G had more CRVs with positive cooperativity, indicating a stronger positive preference for H5_G over H5_A. Interestingly, H5_A had many pairwise interactions with an antagonistic relationship, where two features counteract each other by reducing the overall effect either feature has on the model’s prediction.

The CRV cumulative length and angle plot for position H6 shows a large change in H6_T’s cumulative length in the positive cooperative region, while the cumulative length continues to increase into the antagonistic region of the plot (Fig. 6F). This indicates a strong preference for the H6_T nucleotide at position H6, but also that some pairwise interactions reduce the effect H6_T has on the model’s prediction. We also see covariation between H6_A and H6_G with strong negative cooperative CRVs (Fig. 6F), indicating the model has similar disfavor for H6_A and H6_G. H6_C seemed to also covary with H6_A and H6_G with the model disfavoring H6_C albeit at a much lower cumulative length. This is due to H6_C having shorter CRV lengths in the negative cooperative region (Supplementary Fig. S11C).

Position H7’s CRV cumulative length and angle plot reveals covariation between H7_T and H7_C, with both features showing many CRVs with strong negative cooperativity (Fig. 6G). In this case, many pairwise interactions between H7_T or H7_C and another feature results in negative cooperativity that decreases the model's prediction. Interestingly, H7_A and H7_G covary until midway through the positive cooperative region of the plot where they diverge (Fig. 6G). The CRV cumulative length and angle plots for H7_G showed stronger positive cooperation and antagonism than the other nucleotides at position 7.

At position S1 the CRV cumulative length and angle plots reveal two distinct groups of nucleotides that covary together. S1_A and S1_T covary together with few CRVs within the negative cooperative region of the plot (Fig. 6H). S1_T’s has more antagonistic relationships than S1_A, indicating a similar treatment of S1_A and S1_T with slightly more favor for S1_A by the model. Further, the cumulative length and angle curves demonstrate that S1_G and S1_C covary together, and that both have CRVs with negative cooperativity with a steep slope in quadrant 3 and both have CRVs with antagonism in quadrant 2 and 4 (Fig. 6H). Together the cumulative length and angle plots for positions S1 indicates the model’s disfavor for S1_G and S1_C and the model’s favor for S1_A and S1_T with S1_A being the model’s preference.

The CRV cumulative length and angle plot for S2 revealed strong covariation between S2_A, S2_G, and S2_C. Of the three, S2_G had a higher cumulative length in the negative cooperativity region (Fig. 6I), indicating specifically that the model disfavors S2_G over S2_A and S2_C. The S2_T nucleotide-position did not covary with the other three nucleotide-positions (Fig. 6I). The S2_T has many CRVs that show positive cooperativity, indicating the H4S2 model favors S2_T.

Taken together, the CRV vectors, and their descriptive metrics, give an insightful view on the pairwise contributions of features to a model’s prediction. They give granular perspective to nucleotide bases that the model treats similarly, indicating that those nucleotide bases contribute or function similarly during RAG1/2 mediated V(D)J recombination. It is noteworthy that the CRVs also provide an avenue to identify when the model’s similar treatment of two features’ covariation breaks down.

### Nonamer sequence’s global effects on recombination efficiencies—independent of heptamer sequence

**Figure 7.**
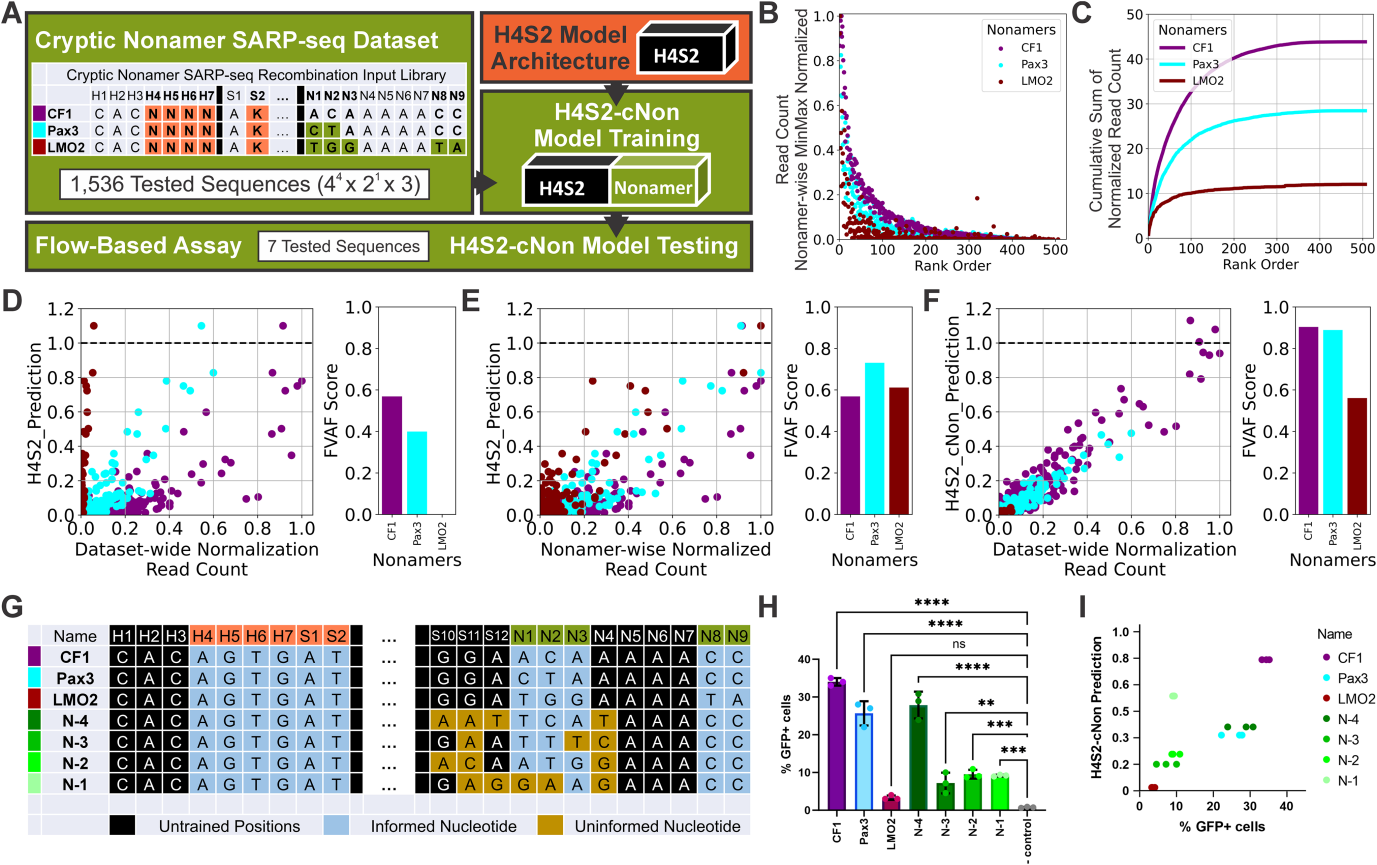
(**A**) Flowchart of training and testing the H4S2_cNon model. The table shows the degenerate 12-RSS sequences in the CF1-, Pax3-, and LMO2- SARP-seq N(H4-H7)K(S2) dataset. (**B**) Recombination activity level of the 12-RSSs in the cryptic nonamer dataset where the 12-RSSs ranked by average read count between the 3 nonamer datasets is plotted on the *x*-axis, and the *y*-axis is the min–max normalized read count for each nonamer subset. (**C**) Cumulative summation of the nonamer-wise normalized read count of each nonamer in the cryptic nonamer datasets. (**D**) Left: The relationship between the H4S2 model predictions and the cryptic nonamer’s dataset-wide normalized read counts, where the entire dataset is min–max normalized without prior grouping. Right, FVAF score for the scatter plot. (**E**) Left: The relationship between the H4S2 model prediction and the cryptic nonamer dataset’s nonamer-wise normalized read counts. Right, FVAF score for the scatter plot. (**F**) Left: The relationship between the H4S2_cNon model prediction and the cryptic nonamer dataset’s dataset-wide normalized read counts. Right: FVAF score for the scatter plot. (**G**) Sequences of externally tested 12-RSS sequences. Black boxes are nucleotide positions that were constant across all training samples and were not encoded as inputs for the model. Light blue boxes are “informed nucleotide” positions that were included in the training samples and were encoded as inputs for the model. Gold boxes are “uninformed nucleotide” positions that were not included in the training datasets, highlighting nucleotides the H4S2-cNon model had not previously seen during training. (**H**) %GFP positive cells measured by the fluorescence-based V(D)J recombination assay with each 12-RSS measured individually. The asterisks denote the P-value range for an ordinary one-way ANOVA with Dunnett’s multiple comparisons test, where ns is not significant; **P < 0.01; ***P < 0.001, and ****P < 0.0001. (**I**) A Pearson r = 0.70 relationship between H4S2_cNon model prediction and the independently measured recombination activity of seven different 12-RSSs.

Next, we explored the impact that the nonamer has on the H4 to S2 region by analyzing a more diversified dataset, the cryptic nonamer SARP-seq dataset: CF1-, Pax3-, and LMO2-N(H4-H7)K(S2) (Fig. 7A). Even with only partial randomization of the 12-RSS, thousands of different 12-RSS substrates from the combined 3 nonamer datasets were recombined in the SARP-seq experiments. The dataset includes CF1, which corresponds to the canonical 12-RSS. Pax3 and LMO2 are the denotations for the nonamers taken from cryptic 12-RSSs, which are found outside the AgR loci and known to have aberrant recombination mediated by mistargeting of RAG1/2 [21]. Plotting the data by the rank order showed that the sequences in the CF1 library typically were better recombination substrates across most of the H4-H7, S2 sequences (Fig. 7B). Sequences in the LMO2 library consistently showed the lowest level of activity across the H4-H7, S2 sequences. This global trend is exemplified when viewing the cumulative read count distributions across the rank order, where there are major differences in the maximum of the cumulative read count distributions (Fig. 7C). The highest maximum cumulative read count was CF1 (above 40), followed by Pax3 (with a maximum over 25), and lastly LMO2 (with the lowest maximum at just above 10) (Fig. 7C). Together, the different nonamer sequences globally scale the recombination efficiencies of the tested 12-RSSs regardless of H4-S2 sequence.

### The absence of sequence coverage from the nonamer stifles H4S2 model prediction

It is currently unknown how other regions of the RSS affect the H4-S2 sequence’s recombination efficiency, although previous results from Hoolehan *et al.* showed that three cryptic nonamer sequences demonstrated little change to DNA selectivity of the H4-S2 region [29]. Even so, the H4S2 model was unable to accurately predict the activity level of the SARP-seq N(H4-S2)K(S2) cryptic nonamer dataset (Fig. 7D). We performed nonamer-wise normalization to control for the global effects the nonamer may have on the H4-H7, S2 sequences, where the read counts for each nonamer are independently rescaled between 0 and 1. With nonamer-wise min-max normalization, the H4S2 model’s performance substantially improved (Fig. 7E), highlighting that normalizing for the global effects of the nonamer sequence may be an adequate method of normalization when data are limited.

To further capture the nuanced interactions between the heptamer and the nonamer, a second neural network model was trained (see workflow in Fig. 7A). The trained H4S2-cNon model predicts the recombination efficiencies from SARP-seq datasets well (Fig. 7F and Supplementary Fig. S6C–E). The nonamer-informed H4S2-cNon model outperformed the H4S2 model's predictions, which lacks any nonamer information (Supplementary Fig. S6F–H) and outperformed the H4S2 model when each nonamer’s global effect was min-max normalized nonamer-wise (Fig. 7E). The observed increase in performance arises from the model capturing the nuanced effects of the nonamer sequence at positions N1 to N3, N8, and N9 to the differing H4-H7, S2 sequences (Supplementary Fig. S6I–K). This indicates that the nonamer’s effect on the heptamer region goes beyond just a global effect, where a specific nonamer motif may exclusively affect certain heptamer motifs.

Seven more variant 12-RSS substrates were independently tested by the fluorescence-based V(D)J recombination assay and the measured recombination efficiencies to the nonamer-informed H4S2-cNon model’s prediction were compared (Fig. 7G–I). The substrates contained variant nucleotides at positions S10-S12 and N4 that are not encoded into nucleotide:position features for the H4S2-cNon model’s input. Additionally, some substrates contained uninformed nucleotides at positions that are encoded as nucleotide:position features, but the particular nucleotide was absent from the training set and was previously unseen by the data. Even with the lack of coverage for some of 12-RSS substrates, the H4S2-cNon model’s predictions show a good correlation between the model prediction and the fluorescence-based measure of V(D)J recombination activity (Fig. 7I). These results demonstrate the H4S2-cNon model’s ability to capture the effects that the heptamer and nonamer sequences have on V(D)J recombination efficiencies. Of note, the H4S2-cNon model overpredicted the N-1 substrate. This substrate happens to also have many nucleotide:position features that the model was not exposed to during training, which further indicates how data with limited sequence coverage can limit the model’s accuracy for those sequences with little coverage.

### Identifying candidate long distance second-order interactions with SHAP values for a nonamer informed H4S2-cNon model

**Figure 8.**
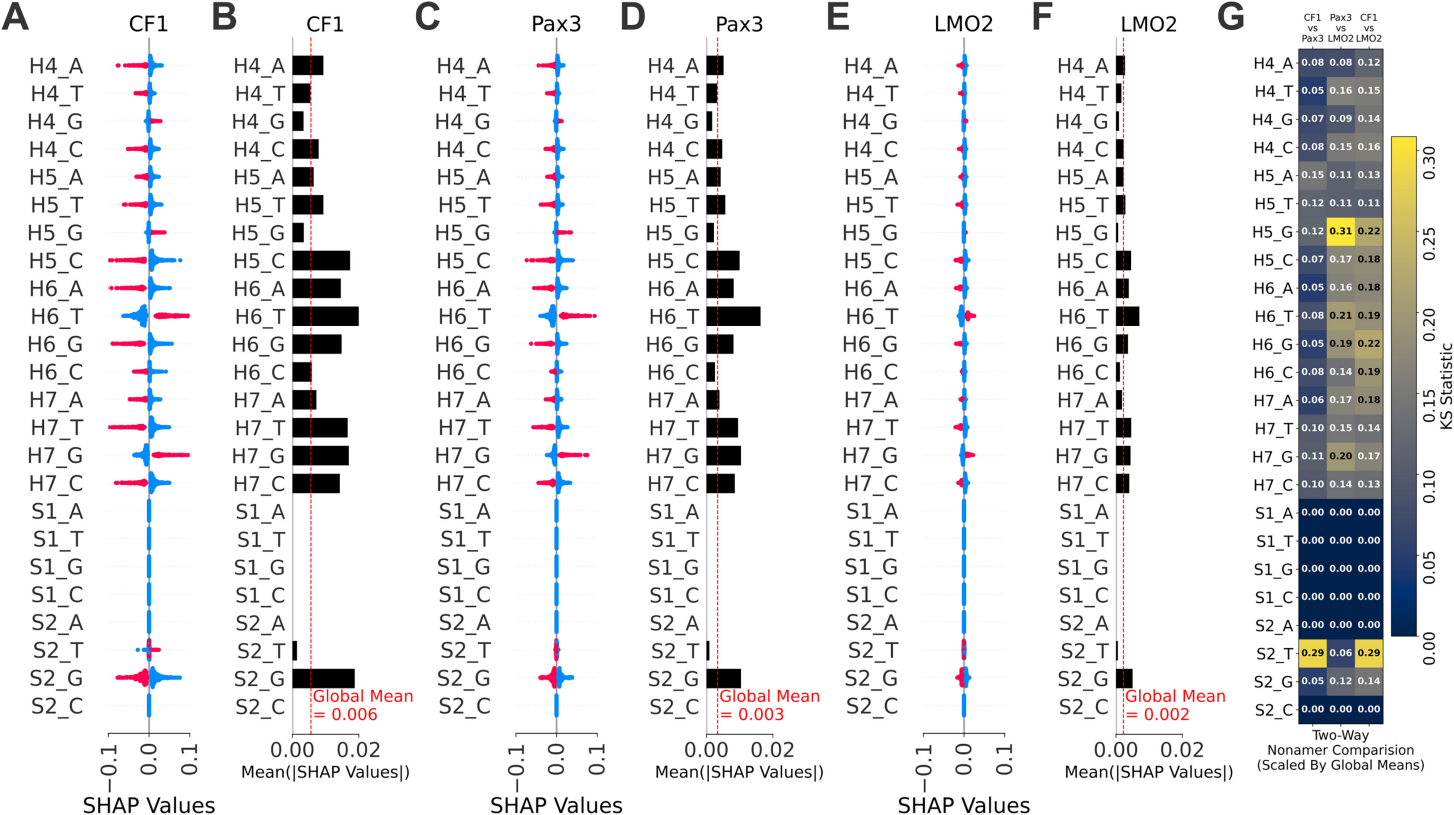
(**A**) SHAP values for the H4-S2 features of the H4S2_cNon model predictions for the CF1 nonamer sequences in the dataset. (**B**) The mean absolute value of SHAP values for the H4-S2 features for the CF1 nonamer sequences in the dataset, i.e. the average nondirectional change on the model's prediction. The dashed red line shows the CF1’s global mean of all H4-S2 features absolute SHAP values. (**C**) SHAP values for the H4-S2 features of the H4S2_cNon model prediction for the Pax3 nonamer sequences in the dataset. (**D**) The mean absolute value of SHAP values for the H4-S2 features for the Pax3 nonamer sequences in the dataset. The dashed red line shows the Pax3’s global mean of all H4-S2 feature absolute SHAP values. (**E**) SHAP values for the H4-S2 features of the H4S2_cNon model prediction for the LMO2 nonamer sequences in the dataset. (**F**) The mean absolute value of SHAP values for the H4-S2 features for the LMO2 nonamer sequences in the dataset. The dashed red line shows the LMO2’s global mean of all H4-S2 feature absolute SHAP values. (**G**) Kolmogorov–Smirnov test statistics for two-way comparisons between two of the three nonamer’s rescaled SHAP values by dividing by each nonamer’s global mean.

As performed above, we used the SHAP library to explain the contributions of each feature on the nonamer informed H4S2 model’s prediction, by calculating the SHAP values for each feature for every sample in the SARP-seq N(H4-H7)K(S2)-cNon dataset. We then separated the SHAP values and visualized their distribution on contributions in separate beeswarm plots (Fig. 8A, C, and E). The ranges for each feature's SHAP values differed under each nonamer, mirroring the cumulative distribution maximum (Fig. 7C). The SHAP values show the nonamer-informed model captures the LMO2’s small activity range and has scaled the feature contributions accordingly. The same can be said for the other nonamers, with CF1 having the features with the largest scale distributions with Pax3 between CF1 and LMO2. This global scaling of the feature’s contributions according to the nonamer is exemplified by the absolute SHAP values for each nonamer, which captures the total impact each nucleotide:position feature has on the model regardless of positive or negative direction (Fig. 8B, D, and F). The global mean of these values provide insights into the scaling used to predict each nonamer H4-S7, S2 sequence. The differences in the global mean indicates that the model depends heavily on the global effects of the nonamer to scale the predictions to the correct activity range.

To obtain a better explanation of the local effects that the H4S2-cNon model utilizes to make its prediction, we compared each feature’s SHAP value distribution of one nonamer sequence to another nonamer sequence (Supplementary Fig. S12). We used the KS test to identify features where the transformed SHAP values distributions are different shapes, and by extension the two nonamer’s effects on a feature may be fundamentally different.

The KS test between the transformed SHAP values reveals LMO2 having the most differences in the distribution’s shapes. For example, KS test comparison between CF1 versus LMO2 or between Pax3 versus LMO2 for H4_T and H4_C showed higher KS metrics than the comparison between CF1 versus Pax3—suggesting little difference between CF1 and Pax3 distributions with larger differences when paired with LMO2. This could indicate a shared long-distance interaction between the nonamer nucleotides that are shared between CF1 and Pax3, but that are not shared with LMO2. The same could be extended to the H5_G, H6_A, H6_T, H6_G, and H7_A, which also show LMO2 having large differences to both CF1 and Pax3. While the KS test of the transformed SHAP values does not provide quantification of the long-distance interactions between the heptamer and nonamer, it does provide a method of identifying candidate long distance interactions that locally exist between certain nonamer motifs and the heptamer, but not other nonamer motifs and the same heptamer.

## Discussion

**Figure 9.**
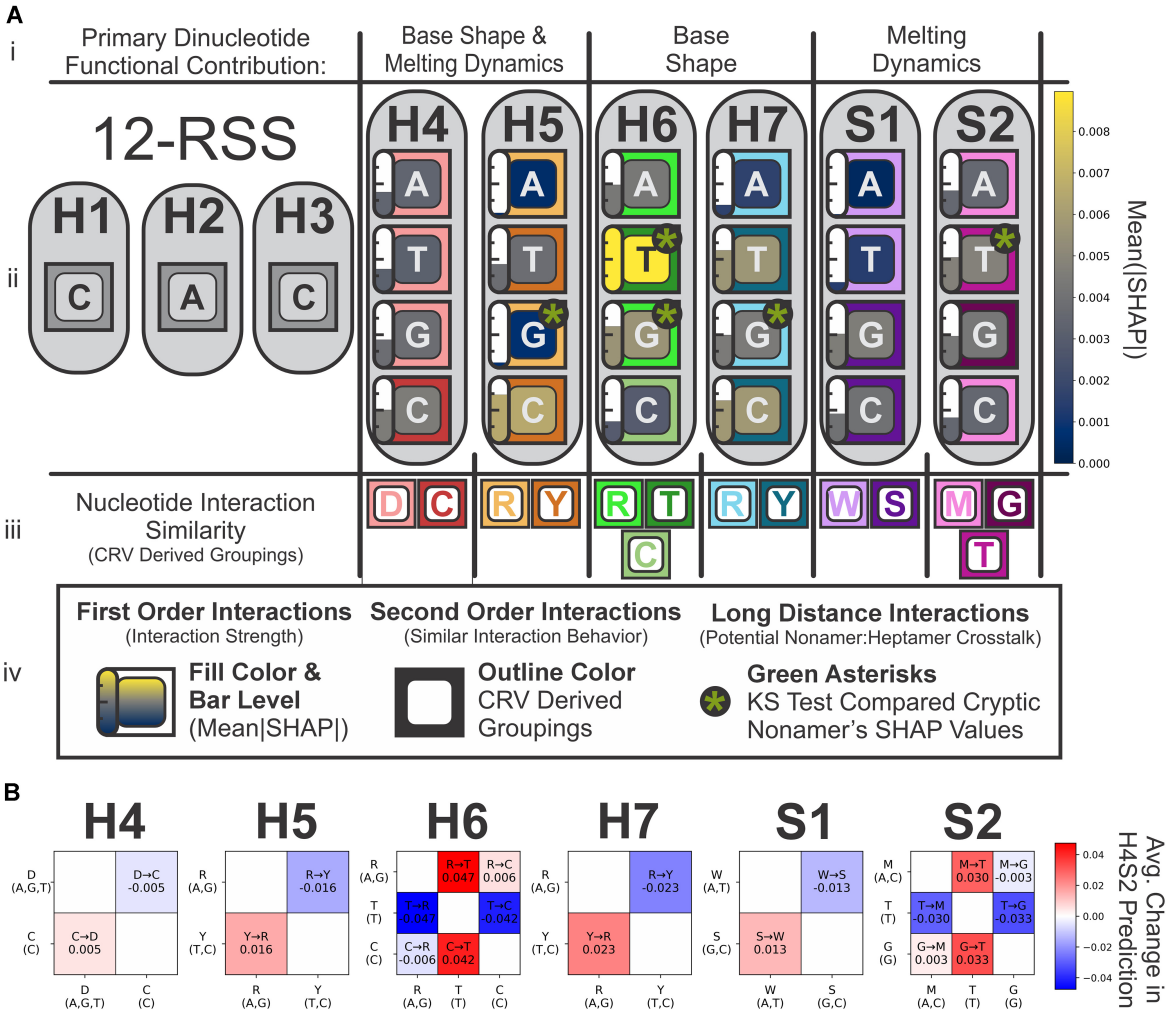
(**A**) Graphical summary of the overall results. (**i**) At top is summarized the major functional contributions of three consecutive dinucleotides (H4-H5, H6-H7, and S1-S2) to V(D)J recombination activity, as described in the text. (**ii**) The four nucleotides are displayed on a grid for every position. The FILL COLOR of each nucleotide and its associated bar's fill level corresponds to the measured strength of the first-order interactions by way of the average absolute change Mean(|SHAP value|) that represents the impact that a nucleotide has on the H4S2 model prediction (see Fig. 5B). The OUTLINED COLOR that frames each nucleotide:position indicates which nucleotides share similar profiles of pairwise interactions with all other input features and that the model does not differentiate from one another. The groupings were derived by way of comparing every position’s four nucleotides and their CRVs by way of cumulative summation (see Fig. 6D–I). The green asterisk indicates potential candidates for local long-distance interactions between the heptamer and nonamer region (see Fig. 8G; where KS metric is ≥0.20). (**iii**) The CRV groupings of the similar computationally behaved nucleotides represented by their corresponding IUPAC ambiguous DNA codes. (**iv**) A legend demarking the location and meaning of the color scheme of each nucleotide:position’s fill color, outline color, and green asterisk. (**B**) A set of heatmaps for each position in the H4-S2 region (see Supplementary Fig. S7A) reduced into minimal categories using CRV second-order interaction groupings (grouped by outline color in Fig. 9A, iv). Each heatmap is the average change in the H4S2 model prediction for the reduced category of possible point mutations at that position by way of simulated mutation of the vertical axis's degenerate nucleotide identity to the horizontal axis’s degenerate nucleotide identity.

In this paper, we propose a neural network H4S2 model that captures the biological underpinnings of the RAG1/2 and 12-RSS interaction at the H4 to S2 positions of the heptamer and adjoining spacer region. By leveraging machine learning and novel explainable AI techniques new insights can be gained on how the RSS sequence affects V(D)J recombination mediated by RAG1/2 recombinase. First, the model demonstrated accurate predictions of recombination efficiency of 12-RSS H4-S2 sequences and a comprehensive, albeit a black box, understanding of how these positions affect recombination activity. Second, we used SHAP to explain the behavior of the H4S2 model beyond a black box model. We explained how the model reaches its recombination efficiency prediction by using first-order interactions between the nucleotide:position features. We expanded the SHAP analysis to quantify and compare pairwise second-order interactions, by comparing the SHAP values of nucleotide:position features. This provided a comprehensive and novel understanding of how the H4S2 model treats nucleotide:position features. Third, we built a nonamer-informed H4S2-cNon model to identify and explore how long distance, second-order interactions between the heptamer and nonamer affect the H4-S2 contributions to the model’s prediction of recombination efficiency. Together, the neural network models and their analysis enabled robust quantification of how nucleotide:position features impact recombination efficiency, which were previously unknown (Fig. 9A).

The graphical summary provides a synopsis of each nucleotide:position feature’s contribution to the H4S2 model’s prediction of V(D)J recombination efficiencies for different H4-S2 sequences. Features that have larger, mean absolute SHAP values, and are the nucleotide:position features with first-order interactions that have higher influences on the model’s prediction (Fig. 9A, fill color). The pairwise comparison of feature SHAP values and their corresponding CRV’s reveals which nucleotides within the same position share similar second-order interactions, as represented by colored square labels that establish the position-wise second-order interaction groupings (Fig. 9A, outline color). By comparing the H4S2-cNon model’s SHAP values for each nonamer, we identified nucleotide:position features that had the highest difference in rescaled SHAP values, with a green asterisk indicating candidate nucleotide:position features with contributions that are altered when paired with specific nonamers (Fig. 9A, asterisk).

The CRV analysis and the cumulative length and angle plots provide a method to group nucleotides that share similar CRVs or similar second-order interactions with all other features. We define the CRV groupings as illustrated in Fig. 9A, outline color. To demonstrate the power of the CRV analysis, the groupings were used to simplify the number of possible DNA point mutations for each position (Supplementary Fig. S7A) down into a minimal number of categories (H4: D/C, H5: R/Y, H6: R/T/C, H7: R/Y, S1: W/S, S2: M/T/G) (Fig. 9B). When a nucleotide was mutated from one CRV group to another, the average change in the H4S2 model prediction was measured, which resulted in strong separations between the minimal categories at each position. The CRV analysis approach essentially identifies nucleotides that function similarly and that function differently within the system. Further experimentation is needed to examine whether these minimal CRV groupings also reflect biochemically distinct behaviors through the RAG1/2-mediated catalytic pathway.

High-resolution structures of RAG-RSS paired complexes have elucidated the backbone-specific and multiple base-specific contacts in the H1-H3 and H4-S2 regions formed during RAG1/2 binding to the 12-RSS [22–24, 50]. Notably, the H1-H3 region in the RAG–RSS complexes is distorted from B-form DNA, with melted DNA and/or base pair stacking misalignments, in a conformational state required for RAG cleavage at the scissile phosphodiester bond between the heptamer and the flanking coding DNA [6, 22, 23]. Here, we demonstrate that H4-S2 sequences with lower melting temperature (*T*_m_) trend toward higher levels of recombination efficiency (Fig. 4). Decreases in Tm indicate an increased dynamic DNA structure, which may lower the energetic barrier of the RSS heptamer to transition to a conformation state required for RAG cleavage activity. The highly bent spacer region may further provide torsional energy that propagates through the H4-S2 region toward the H1-H3 region to facilitate DNA melting bubble formation. Certain global properties, such as decreased *T*_m_, that favor increased DNA distortions may explain RAG1/2’s capability to cleave thousands of highly diverse RSS sequences.

Based on the combination of methods used here, we propose that three consecutive dinucleotides in the RSS heptamer and adjoining spacer make distinct contributions to recombination activity (summarized at the top of Fig. 9A). First, the H6-H7 dinucleotide makes a dominant contribution to recombination activity, as emphasized by the CRV analysis (Fig. 6F and G). The dominance of this dinucleotide can be attributed to base shape (Y-R at H6-H7) and also some of the few base-specific interactions (particularly to H6) in RAG–RSS complexes, as shown by high resolution structures [23, 24]. The S1-S2 dinucleotide contributes to recombination activity of all heptamer sequences through a clear dependence on *T*_m_ (Fig. 4D and E). Further, the CRV analysis confirms that the preference for weak base pairs (A:T) at these positions leads to improved recombination activity. The contribution to recombination activity of the H4-H5 dinucleotide is apparently smaller than either the H6-H7 or S1-S2 dinucleotides; however, it may play differing roles depending on sequence content of neighboring bases. First, the thermal stability of H4-H5 appears to function exclusively with RSS heptamers that contain G at position H6, where weak base pairs at H4-H5 would render such RSSs more likely to be utilized in V(D)J recombination (Fig. 4E). Second, CRV analysis demonstrates that C in either H4 or H5 is disfavored for recombination activity (Fig. 6D and E). In this case, RSSs that contain identical sequences in H6-S2 will have the lowest activity with C-C at H4 and H5 compared to other base combinations. Together, this model illustrates the unique contributions that dinucleotides within the RSS heptamer/adjoining spacer have on recombination activity (Fig. 9A).

These studies are based on SARP-seq experimental results that used catalytically essential core regions of RAG1/2. However, the fluorescence-based assays performed in this study were with full-length RAG2, with our results consistent with the lack of any DNA-binding motifs within the noncore RAG2 region. Even though sequence-specific interactions between the noncore RAG regions with RSSs have not been detected, the noncore regions may function in stabilizing the RAG1/2 complex with the paired 12/23 RSSs [3, 51–53]. Machine learning techniques and approaches used in this paper will be valuable in distinguishing the full extent of full-length versus core RAG1/2 on recombination efficiencies of RSS variants, once sufficient data are available. Further, the SARP-seq results were performed using a plasmid-based assay, in which DNA sequence-specificity is the primary driver for differential recombination activity between RSSs of varying sequences. By contrast, the context of the AgR loci has multiple factors that can affect V(D)J recombination efficiency on the RSSs including RAG-recruitment through chromatin interactions, structural conformations of the loci, and locus-specific promoter and enhancer elements [15–18, 47, 54–56]. In such complex systems, we argue that it is essential to elucidate how the fundamental principles of DNA sequence affects V(D)J recombination. As an example, single-nucleotide polymorphisms (SNPs) have been identified in RSSs in humans, and in some cases have been shown to have deleterious effects on the repertoire and adaptive immune response [57–59]. As the SNPs for a given RSS would presumably share the same genetic, chromatin, and regulatory environments, any difference in recombination would stem from the sequence differences. The approaches and models used here could be leveraged to differentiate if newly identified SNPs would significantly affect RSS usage and alter the AgR repertoire of affected individuals or populations.

Our results provide a detailed explanation of how the H4S2 model and H4S2-cNon model utilized the nucleotide:position features to make a sequence-based prediction of the V(D)J recombination efficiencies. The models have demonstrated robust prediction of the V(D)J recombination efficiencies by validation with a flow-based assay completely independent from the training SARP-seq datasets (Fig. 5E and Fig. 7I). The H4S2 models also effectively predicted unseen experimental replicates (Fig. 2C and D, and Fig. 7D), with only one sequence (AGTG|CT) being overfit by the H4S2 model. This bias was inherited from the data underlying the SARP-seq N(H4-S2) experiment (Supplementary Fig. S5). However, the use of dropout regularization helped to ensure that no single example or set of features are relied on to make the model’s prediction, and we presume the thousands of correct samples with nearly zero residual error and standard deviation across experimental replicates compensate for the few discrepancies seen in the H4S2 model. Considering both of the models’ performance and generalizability, the interworking’s of H4S2 model and H4S2-cNon model may by extension reflect the biological underpinnings that govern the H4-S2 region’s sequence-based interactions and how they influence recombination efficiencies. The CRV analysis highlighted nucleotide:position features that were being treated similarly by the H4S2 model (Fig. 6D–I and Fig. 9A). While the H4S2 model captures the local interactions within the H4-S2 region, future experiments expanding our sequence coverage in the spacer and nonamer are needed. Further, the H4S2-cNon model identified potential nucleotide:position features that have their local interactions within the H4-S2 region altered when paired with specific nonamer sequences (Fig. 8G and Fig. 9A, green asterisk). While limited number of candidate long-distance interactions were identified, the alterations were not prominent across all nucleotide:positions and typically seemed to be limited to only certain nonamer sequences, However, further computational development is needed to provide quantification of the strength of the long-distance interactions, as well as the caveats on which they operate.

Together this work provides a more granular perspective of the impact for each nucleotide:position in the heptamer and a joining spacer, as well as potential synergistic effects with the nonamer. The developed H4S2 model captures the complex interaction within the H4-S2 region to predict H4-S2 sequence’s recombination efficiencies. Quantifying the SHAP values’ first-order interactions and the pairwise SHAP values’ second-order interactions provide a unique perspective on how nucleotide:position features interact with one another. There are limitations still to be resolved, including that the SARP-seq experiment maintained a constant canonical 23-RSS of the 12/23 paired RSSs, which limits our perspective of interactions between the 12- and 23-RSSs. Even more so, the diversity of the 12-RSS alone is overwhelming and can quickly limit experimental feasibility. For example, the RSS consists of the heptamer, spacer, and nonamer, as well as a coding flank sequence 5′ of the heptamer. Although conducted under different experimental formats, the other regions of the RSS—the coding flank [60–62], the nonamer [25, 63], and the spacer [25, 63–65]—have previously been shown to affect V(D)J recombination activity. Notably, Hoolehan *et al.* tested three different coding flanks, the results found that the coding flank did not impact the relative selectivity within the H4-S2 region and that the coding flank could define a coarse level of recombination regardless of the RSS’s sequence content. We discuss this coarse level as a global scaling that can affect all RSS sequence’s recombination level, and as such, this coarse level is presumed to be refined to the experimentally measured values by the influence of relative selectivity within the H4-S2 region [29]. This global scaling—without affecting the relative selectivity of the H4-S2 region—was also seen in the cryptic nonamer dataset. Our H4S2-cNon model’s SHAP values also confirmed the large global scaling of the nonamer region to affect H4S2 region in a sequence independent manner (Fig. 8A–E). Beyond the global scaling, the H4S2-cNon model suggests the nonamer region can affect the H4S2 region in a sequence dependent manner, albeit with small, nuanced, long-distance interactions (crosstalk) that fine-tunes recombination activity (Fig. 8G). Future high-throughput V(D)J recombination experiments that expand our sequence coverage in the spacer, nonamer, and coding flank are needed to identify and quantify the interactions within each region and how they influence recombination and to characterize the potential crosstalk between regions. Further efforts should be made to determine the potential of using global scaling factors in place of full interaction characterization when data is limited. Such use of global scaling factors could accelerate the generation of a predictive model that has full sequence coverage of the entire RSS (Fig. 7E).

Understanding the interactions between RAG1/2 and its RSS substrates presents a challenging experimental and computational problem that requires novel experimental design and innovative analytical techniques. Here, our work specifically focused on the H4-S2 region of the 12-RSS, where we identified and quantified previously unknown interactions to uncover the biological underpinnings that the heptamer and adjoining spacer has on RAG1/2-mediated V(D)J recombination efficiencies. Additionally, global and local effects of candidate long distance interactions between the heptamer and nonamer were identified.

## Supplementary Material

gkaf551_Supplemental_Files

## Data Availability

The datasets used in this paper can be accessed in Supplementary Dataset S1 or GitHub repository. Individual linear regression results can be accessed in Supplementary Dataset S2. Jupyter Notebooks were used to preprocess data, to train, and to evaluate our different models, and these notebooks can also be accessed through the Zenodo Repository (https://doi.org/10.5281/zenodo.14903647) or the GitHub repository (https://github.com/A-Blue-Jay/Building-a-Neural-Network-Models-Notebooks).
