# Supplementary material for: Building a neural network model to define DNA sequence specificity in V(D)J recombination
Source: Nucleic Acids Res. 2025 Jun 23;53(12):gkaf551. doi: 10.1093/nar/gkaf551 (PMC12205992; doi:10.1093/nar/gkaf551)
Supplement: gkaf551_Supplemental_Files [file gkaf551_supplemental_files.zip › Supplementary_Material_Draft_27_052325_MBS.docx]

SUPPLEMENTARY MATERIAL

**RIC score algorithm PERL script**: A folder containing the PERL script used to calculate the

RIC score for the 12-RSS RIC score. for completeness the folder also includes the script and

files for the 23-RSS RIC score.

**Supplementary Dataset 1**: An excel that contains the SARP-seq N(H4-S2) and the SARP-seq

SARP-seq N(H4-H7)K(S2) datasets used in model training. The Train, Validation, and Test

assignments used for k-fold cross validation.

**Supplementary Dataset 2**: The regression results for all 4-mer sequences at all alignments to

measure the dependence of melting temperature, sequence content and alignment on H4S2

model prediction.

**S9_res**: A high-resolution version of Supplementary Figure S9.

[SUPPLEMENTARY METHODS 2](#_Toc194014272)

[Introduction to Neural Networks 2](#_Toc194014273)

[Input One-Hot Encoding 2](#_Toc194014274)

[Output Normalization 2](#_Toc194014275)

[Model Training 2](#_Toc194014276)

[Model Optimization with K-fold Cross Validation 3](#_Toc194014277)

[Definitions: 4](#_Toc194014278)

[SUPPLEMENTARY FIGURES 6](#_Toc194014279)

[Supplementary Figure S1. 6](#_Toc194014280)

[Supplementary Figure S2. 7](#_Toc194014281)

[Supplementary Figure S3. 8](#_Toc194014282)

[Supplementary Figure S4. 9](#_Toc194014283)

[Supplementary Figure S5. 10](#_Toc194014284)

[Supplementary Figure S6. 11](#_Toc194014285)

[Supplementary Figure S7. 12](#_Toc194014286)

[Supplementary Figure S8 13](#_Toc194014287)

[Supplementary Figure S9. 14](#_Toc194014288)

[Supplementary Figure S10. 15](#_Toc194014289)

[Supplementary Figure S11. 17](#_Toc194014290)

[Supplementary Figure S12. 18](#_Toc194014291)

[Supplementary Table S1. 19](#_Toc194014292)

[REFERENCES 20](#_Toc194014293)

SUPPLEMENTARY METHODS

Introduction to Neural Networks

Neural Networks are complex nonlinear mathematical formulas that utilize input data to generate an output prediction. The power of neural networks comes from the ability to identify and leverage many different complex, often hidden, nonlinear relationships between the input data (1). The model then trains over many examples to learn an optimal way to use these nonlinear relationships while calculating a prediction output.

Input One-Hot Encoding

To perform this calculation, the input data must first be encoded into a number, so that it can be used in the model’s mathematical calculations. Further explanations on encoding are included in the detailed primer by Gupta et al. (2). DNA nucleotides are nominal categories, where there is no specific ordering of the nucleotides. Further, since only one nucleotide can occupy a given position along a DNA sequence, we can assume only one nucleotide is present at a position and all other nucleotides are absent at a position. One-hot encoding separately encodes each possible category as separate features with these assumptions (**Supplementary Figure S1A**). Each position (pos) along the DNA sequence can be one of four nucleotides (A, T, G, C). One-hot encoding creates a four-bit representation for a position and makes four binary features (pos_A, pos_T, pos_G, pos_C) that can have a value of 0 or 1. If the nucleotide is A, the value of pos_A would be 1 while all other Nucleotide:Position features pos_T, pos_G, and pos_C would be 0 (**Supplementary Figure S1A**). This encoding contains no *a priori* bias towards any specific nucleotide, facilitating the model’s ability to identify complex relationships between the input nucleotides.

Output Normalization

An important step of preprocessing data before training a neural network model is the normalization of input and output data. Here we use min-max normalization, which is a linear transformation of the data, where zero is the new minimum and one is the new maximum of the normalized distribution. Normalization isn’t needed for the DNA one-hot encoded features, as they are binary with a range of zero to one. However, for our output target the training dataset is a continuous variable that ranged from 0 to ~18,000 read counts in the iSeq1 experimental replicate. Min-max normalizing the output target to a similar scaling to the inputs will improve model training, as the incremental changes that are needed throughout training are easier when the scale is similar (3).

Model Training

A fully connected dense neural network was used, which is arranged in layers of nodes. Each node implements a non-linear transformation of the state of the previous layer. Specifically, the node first computes a weighted sum of the previous layer state (plus a bias term), and then applies a non-linear scalar function. (**Supplementary Figure S1B**) (1). As the outputs of one layer are the inputs for the next, each node in one layer has access to the same information. Because the weights (parameters) are different for each node in the layer, they can each identify different relationships contained in the state of the previous layer. Together these unique interactions form the complex nonlinear relationships that the model utilizes to make its prediction (4,5). Through training of numerous examples, each node’s weights and biases are updated to optimize performance. This is done first by initializing the weights randomly, measuring the model’s performance with a loss function, followed by a backpropagation step. Backpropagation is a gradient descent technique that can determine the direction to change the weights/biases that will best make incremental improvement in loss. The weights and biases are adjusted accordingly, and the cycle is repeated again (**Supplementary Figure S1C**) (3). By iteratively updating the weights through training, the model builds a mathematical representation of the input data to the output. The iterative training continues until early-stopping halts training, when the performance on a validation subset stops improving. In short, a neural network uses a complex arrangement of interconnected nonlinear functions, to train a mathematical representation that learns complex interactions between the input features and through iterative improvement can use those interactions to accurately calculate an output.

Model Optimization with K-fold Cross Validation

Hyperparameters are tunable, user-set values used in model training, which can affect the performance and quality of the model. The value of these hyperparameters can alter the performance of a model (**Supplementary Figure S4**) (6). An important step in building a neural network is optimizing the hyperparameters; however, this requires repeatedly training a model using different values for each hyperparameter to find an optimized configuration of each different hyperparameter. The model architecture has hyperparameters that include the number of layers, the number of nodes within each layer, and which nonlinear activation function is used within each node’s internal logic. This repetitive training, however, raises concerns of overfitting the training dataset (7), which is when the model memorizes the data in such a way that it cannot generalize to unseen data and is uninformative to that unseen data. For model training, the required hyperparameters can include the loss function used to monitor the model’s performance through the training process, the learning rate which is the size of the step that is taken during backpropagation to iteratively improve the weights and biases, and the number of epochs or the number of iterations of backpropagation that are performed to train the model (8). We used a step wise approach to optimization where we varied one hyperparameter at a time until all hyperparameters were optimized (**Supplementary Figure S3A-B and S4**). Early stopping was used to monitor the model’s training on a validation dataset and detected when the model started to overfit to the training dataset and thereby halted the training early (**Supplementary Figure S1C**). Dropout regularization was used to silence randomly selected nodes throughout the training process in order to prevent overreliance on any one set of nodes or interactions to make the prediction calculation (9).

A powerful tool used in our optimization was k-fold cross validation. In brief, k models are each trained on a unique combination of partitioned data. For k-fold cross validation, first the data set must be split into equally sized folds. As our training dataset has an asymmetric distribution (**Supplementary Figure S3C**), we used stratified sampling to generate representative folds, to ensure that under- and over- represented samples are fairly represented. The dataset was split into 20 representative folds where each fold shared the same distribution of samples as the original dataset (**Supplementary Figure S3D**). Second, the folds were then all combined into 20 unique rotations where 18 folds are used for model training, one fold is used for model validation, and one fold is used for model testing. This is repeated 20 times, making 20 unique rotations of the data such that every fold is only used once as the validation subset in one of the 20 models and once as the testing subset in one of the 20 models. The 20 rotations were used to train 20 models on different hyperparameters selections. For every hyperparameter choice, the performance distributions of the 20 models was compared and the hyperparameter value was decided by visually maximizing the performance of both training and validation subsets, while avoiding hyperparameter choices that had large differences between the performance means of the train and validation subset.

Definitions:

- **Backpropagation**
  1. A gradient descent algorithm in which the weights and biases are updated to improve the model’s performance with respect to the training data set according to some performance metric or loss function (8).
- **Dropout Regularization**
  1. A regularization technique used to prevent overfitting by silencing random nodes from the network during individual training steps to prevent overreliance on any given set of nodes within the network (9).
- **Early Stopping**
  1. A regularization technique used to prevent overfitting by monitoring the model’s performance of the validation set to detect, and then halt training when overfitting is detected.
- **Epochs**
  1. One iteration/ round of improving the weights and biases of the network through backpropagation. Often training limits the number of epochs to halt training after the set number of training steps taken.
- **Hyperparameters**
  1. Tunable user-set parameters that impact model training and performance. Often poses optimization challenges to find the best configuration of multiple parameters to train a model.
- **Fold**
  1. A split of the data.
- **k-fold Cross Validation**
  1. A method to split data into k-folds, that are rotationally combined into unique k-rotations. Each rotation is used to train a separate model and together their performances can be statistically compared.
- **Rotation**
  1. A unique combination of k-folds. Each rotation contains k-2 folds for training, 1-fold for validation, and 1-fold for testing. We rotate through the folds to define the k rotations such that each fold is used as a validation subset and as a testing subset only once.
- **Learning Rate**
  1. The size of the steps taken when improving the weights and biases by backpropagation.
- **Loss Function**
  1. The function used to measure the error of the model during training. The goal of backpropagation is to select model parameters that minimize this loss.
- **Nodes** (in a Neural Network Layer)
  1. A mathematical unit of a neural network. Uses a weighted summation followed by a nonlinear transformation to generate an output value to pass to the next layer of nodes in the network.
- **One-Hot Encoding**
  1. An encoding technique that converts categorical features into multiple binary features.
- **Overfitting**
  1. Adjusting the weights and biases of the model such that they can readily predict the training set well, but are unable to predict unseen datasets.
- **Stratified Sampling**
  1. A method of sampling equal representations from each binned category, by using the population of each binned category. Used to split a dataset into k representative folds.
- **Weighted Summation**
  1. A linear function that scales each input separately (by a coefficient or slope) and adds the scaled values together (a bias or intercept).
- **Weights and Biases**
  1. Are algorithmically selected models’ parameters that are used by the model’s internal logic to scale and calculate the node’s inputs and outputs of the model. These values are iteratively improved/selected through training and by backpropagation.

SUPPLEMENTARY FIGURES


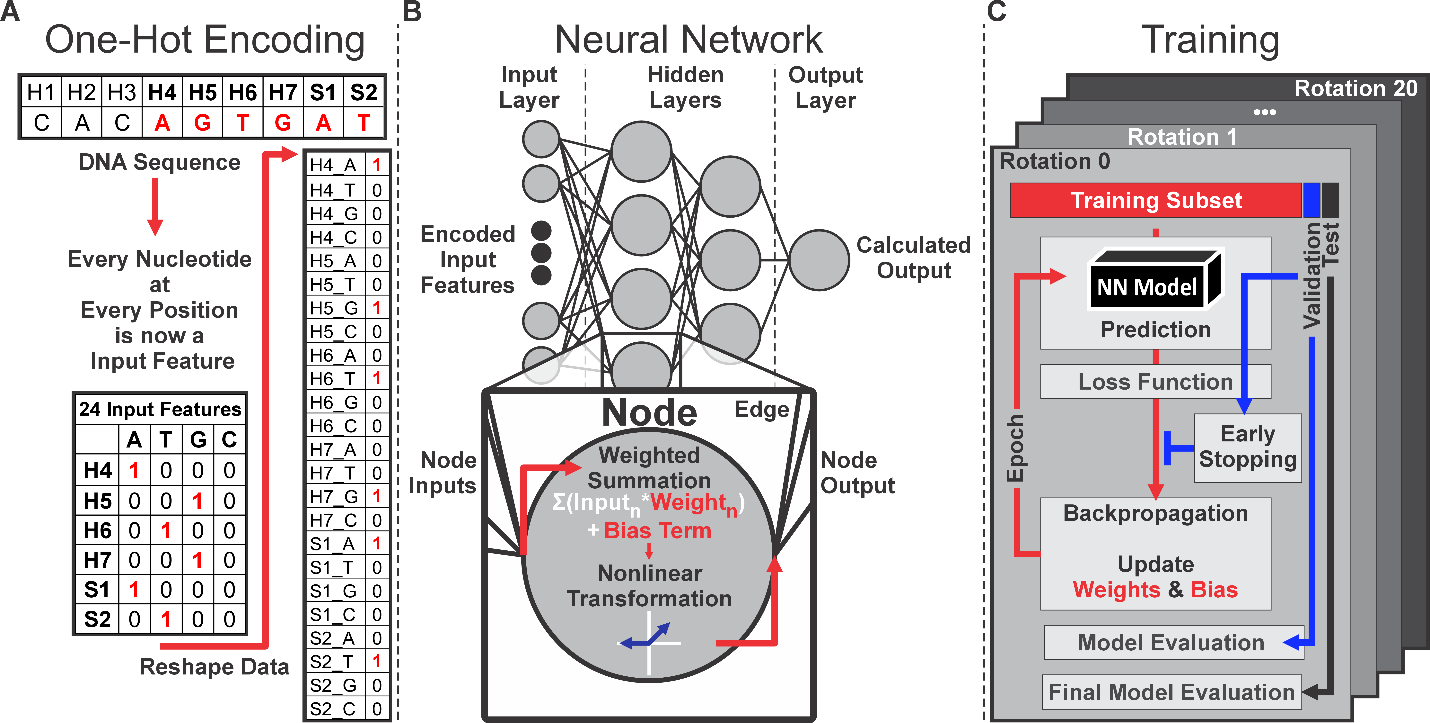


Supplementary Figure S1. **A)** A representation of one-hot encoding, where nucleotides are converted into a 4-bit binary representation for each position and then reshaped so each Nucleotide:Position feature is a separate input feature and can be mathematically independent of one another. **B)** A representation of the structure of a dense fully-connected neural network, where the network is arranged into layers of nodes. The nodes in the input layer are the encoded input features that connect to every node in the hidden layers. The hidden layer’s nodes consist of a nonlinear mathematical function. The previous layer’s outputs undergo a weighted summation and nonlinear transformation into the node’s output, where it is fed into the next layer as an input. This interconnectedness of the nodes in each layer and their nodes allows high-order nonlinear relationships and boundaries to be created that help to predict the correct output. The weights and biases of each node allows each node to focus on different aspects of these relationships. However, the interconnectedness also creates the black box problem where it’s difficult to understand how a model reaches its output decision. **C)** A flowchart on the step to neural network model training, where a dataset is split into a training, validation and test subset and the model’s weights and biases are initialized at some random state. First the model predicts the training subset and measures its predictive loss, usually a measure of the model’s error. Then backpropagation calculates the direction in which to update all weights and biases to reduce the model’s loss. This process is iteratively repeated until a set number of Epochs (cycles) or when early stopping detects overfitting by monitoring the validation’s subset loss each iteration. During model development and hyperparameter selection, an evaluation of the validation subset is conducted. Only after complete optimization should the test subset be evaluated. For k-fold cross validation this process is repeated k times for different rotations of the data.

Supplementary Figure S2. Plasmid-based V(D)J recombination assays **A)** An overview of the SARP-seq assay (using the N(H4-S2) substrate as an example), which includes generating the plasmid input library, transfecting the plasmid library into RAG1/2-expressing cells, culturing the cells for up to 72 hrs to allow V(D)J recombination to proceed, harvesting plasmid DNA, amplifying the signal joints by PCR, and analyzing the sequences used in V(D)J recombination by next generation sequencing (NGS). **B)** Fluorescence-based V(D)J recombination assay. Single sequence plasmid substrates are transfected into RAG1/2-expressing cells and cultured for up to 48 hrs. V(D)J recombination results in the inversion of the GFP gene into a proper orientation for transcription. The cell population is analyzed by flow cytometry to determine the percentage of cells in which a plasmid substrate has undergone a V(D)J recombination event.


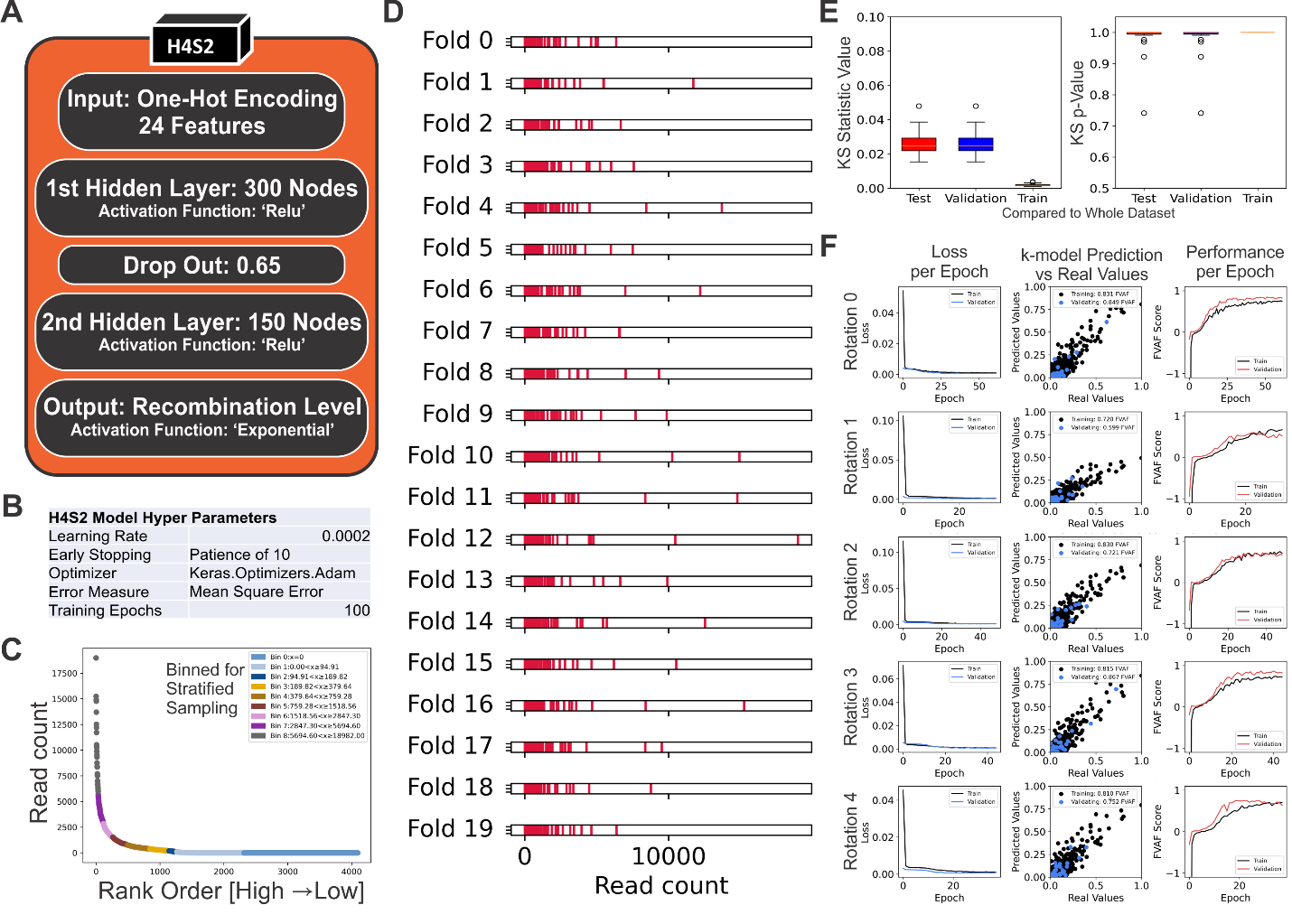


Supplementary Figure S3. **A)** H4S2 NN model architecture and **B)** hyperparameters. **C)** Plot of SARP-seq experimental read counts for recombined RSS substrates from the N(H4-S2) iSeq1 dataset versus the 12RSS sequences in high to low rank order. Bins used in representative stratified sampling are shown in the colors indicated. Using the percentile of the dataset’s read counts as bin thresholds ('0.0', '0.005', '0.01', '0.02', '0.04', '0.08', '0.15', '0.3', and '1' percent of the maximum read count) the continuous distribution was categorized to be compatible with stratified sampling. **D)** Plots depicting each of the 20 dataset splits using the stratified sampling which are rotated through for cross validations. **E)** Kolmogorov–Smirnov test between each rotation’s training, validation, and testing splits versus the entire dataset, confirming representative sampling. LEFT shows KS statistic and RIGHT shows the p-value of the performed tests. **F)** Five representative rotation trainings for 20-fold cross validation. LEFT panels, mean squared error, loss function of the training epochs; MIDDLE panels, the plot of the prediction values for the trained models versus the target Min-Max normalized read counts; and RIGHT panels, Fraction of Variance Accounted For (FVAF) of the training epochs.


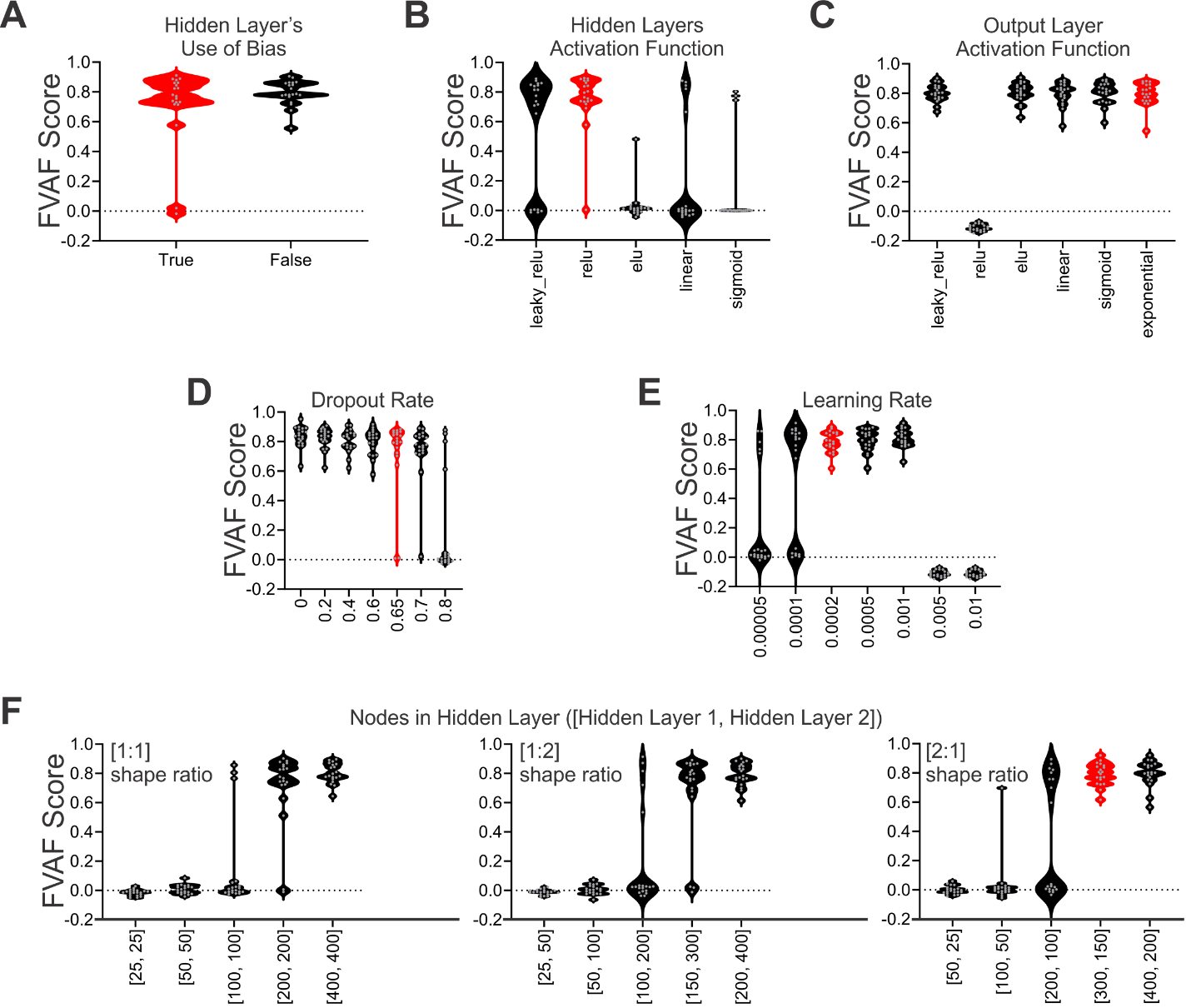


Supplementary Figure S4. Post-hoc survey of the k-fold model’s hyperparameters and architectures after optimizing the H4S2 neural network model to ensure a local minimum. While holding the already optimized hyperparameters and architectures constant, each hyperparameter was varied to ensure the optimal choice was used. Plots comparing **A)** the use of bias in the hidden layers, **B)** the hidden layer’s activation function, **C)** the output layer’s activation function, **D)** the model’s dropout rate, **E)** the model’s learning rate, and **F)** the model’s hidden layers size and shape. The hyperparameter value selected to be used in H4S2 model and later H4S2-cNon model is colored red.


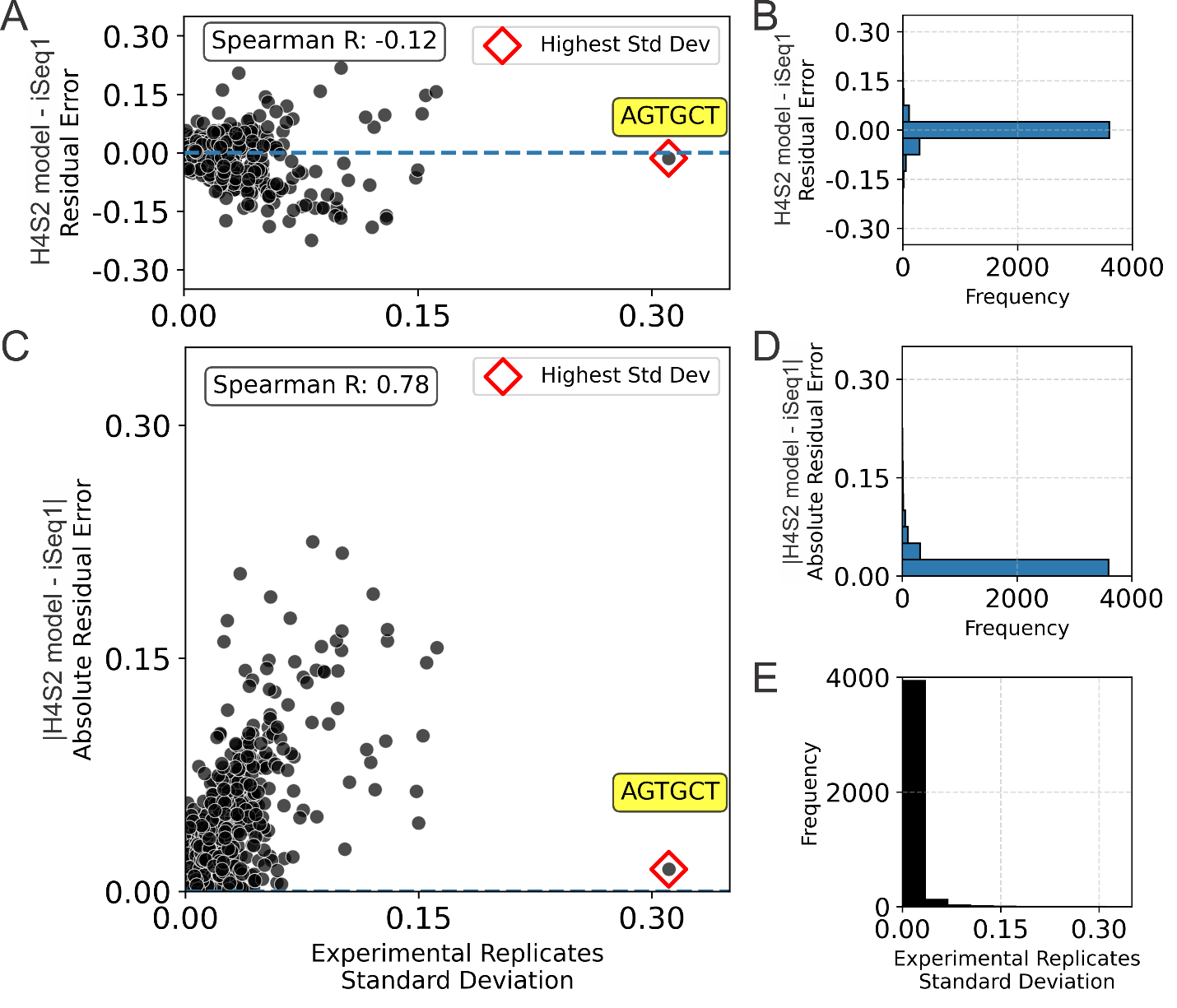


Supplementary Figure S5. **A)** A scatter plot of the training iSeq1 dataset’s residual error (H4S2 model - iSeq1) compared to the standard deviation seen in the training (iSeq1) and two test (iSeq2 and miSeq1) datasets, with a Spearman rank order correlation -0.12 *ρ*. Blue line represents zero residual and no model error. Highlighted is the H4-S2 sequence with the highest deviation across experimental replicates (AGTG|CT). **B)** Histogram distribution of the training iSeq1 dataset’s residual errors. **C)** A scatter plot of the train iSeq1 dataset’s absolute residuals (|H4S2 model – iSeq1|) compared to the standard deviation across the three experimental replicates, with a Spearman rank order correlation 0.78 *ρ*. Highlighted is the H4S2 sequence with the highest deviation across experimental replicates (AGTG|CT). **D)** A histogram distribution of the iSeq1 absolute residual error. **E)** A histogram distribution of the standard deviation across the three experimental replicates.


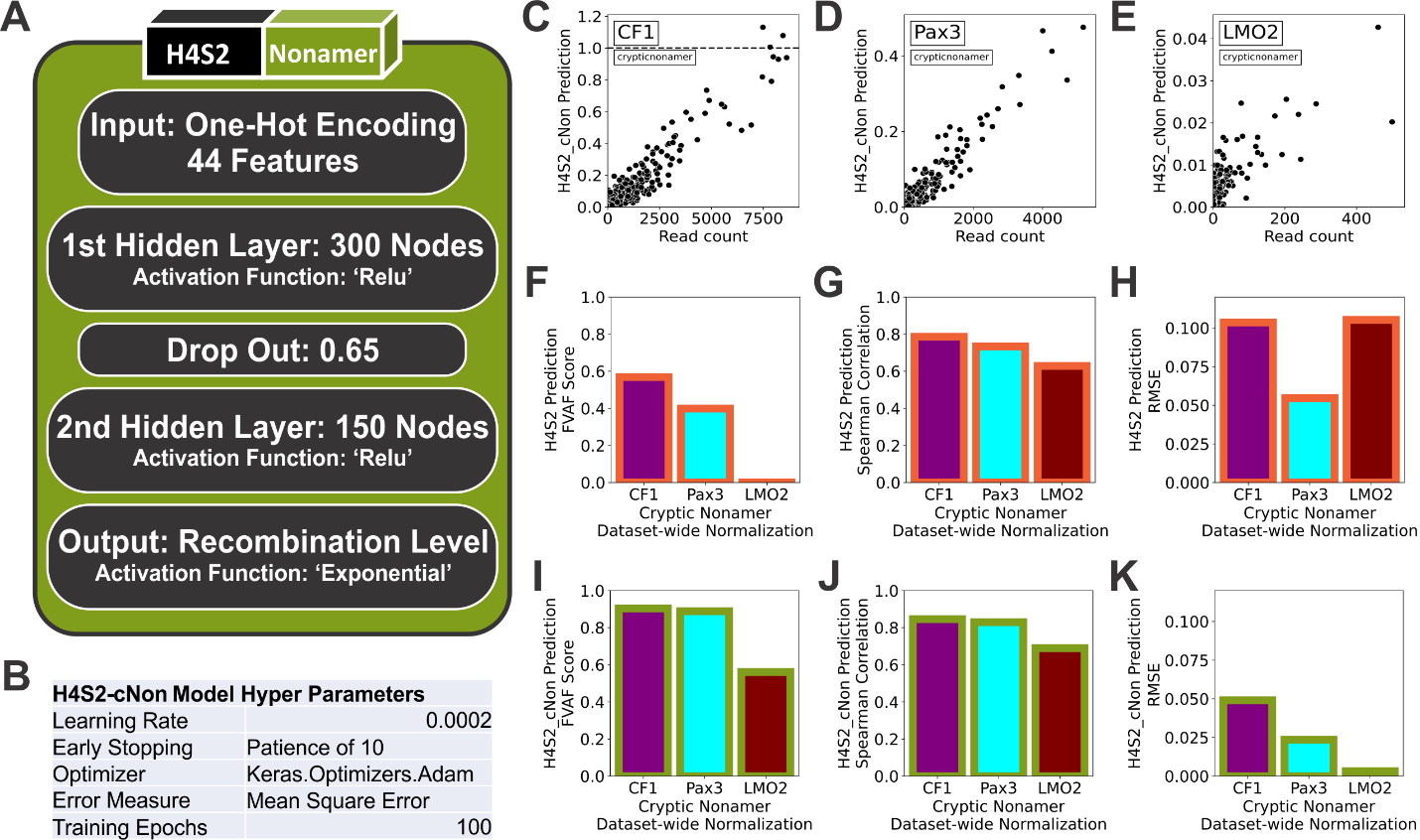


Supplementary Figure S6. **A)** H4S2_cNon model architecture and **B)** hyperparameters. The relationship between the H4S2_cNon model and the read count for the 12-RSSs in the cryptic nonamer dataset with the **C)** CF1 nonamer, **D)** the Pax3 nonamer, and **E)** the LMO2 nonamer. **F)** Fraction of Variance Accounted For (FVAF) of H4S2 model predicting the recombination level of each nonamer subset. **G)** Spearman Rank Order Correlation of H4S2 model predicting the recombination level of each nonamer subset. **H)** Root mean squared error of H4S2 Model Predicting the recombination level of each nonamer subset. **I)** Fraction of Variance Accounted For (FVAF) a performance metric of H4S2_cNon model predicting the recombination level of each nonamer subset. **J)** Spearman Rank Order Correlation a performance metric of H4S2_cNon model predicting the recombination level of each nonamer subset. **K)** Root mean squared error, and error metric of H4S2-cNon Model Predicting the recombination level of each nonamer subset.


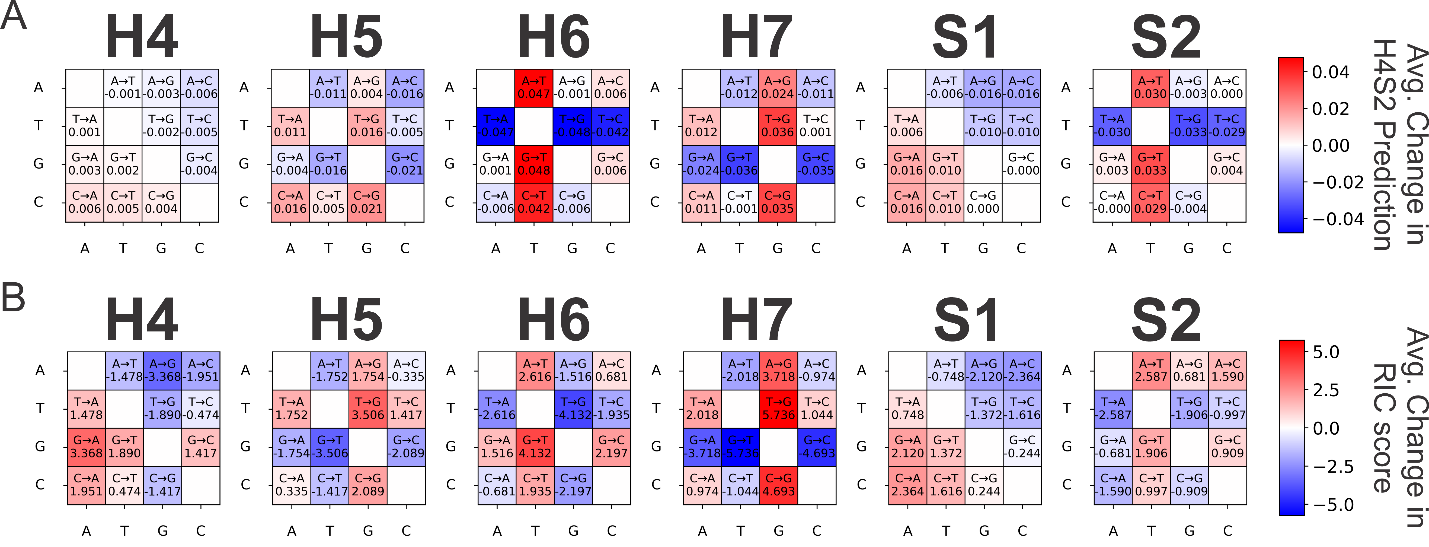


Supplementary Figure S7. A set of heatmaps of each position of the H4-S2 region. **A)** The average change in H4S2 model prediction with every theoretically possible single nucleotide polymorphism (SNP) at each position. In each heatmap the vertical axis’s nucleotides are simulated to mutate into the horizontal axis’s nucleotides. **B)** The average change in the RIC score for every possible SNP at each position.


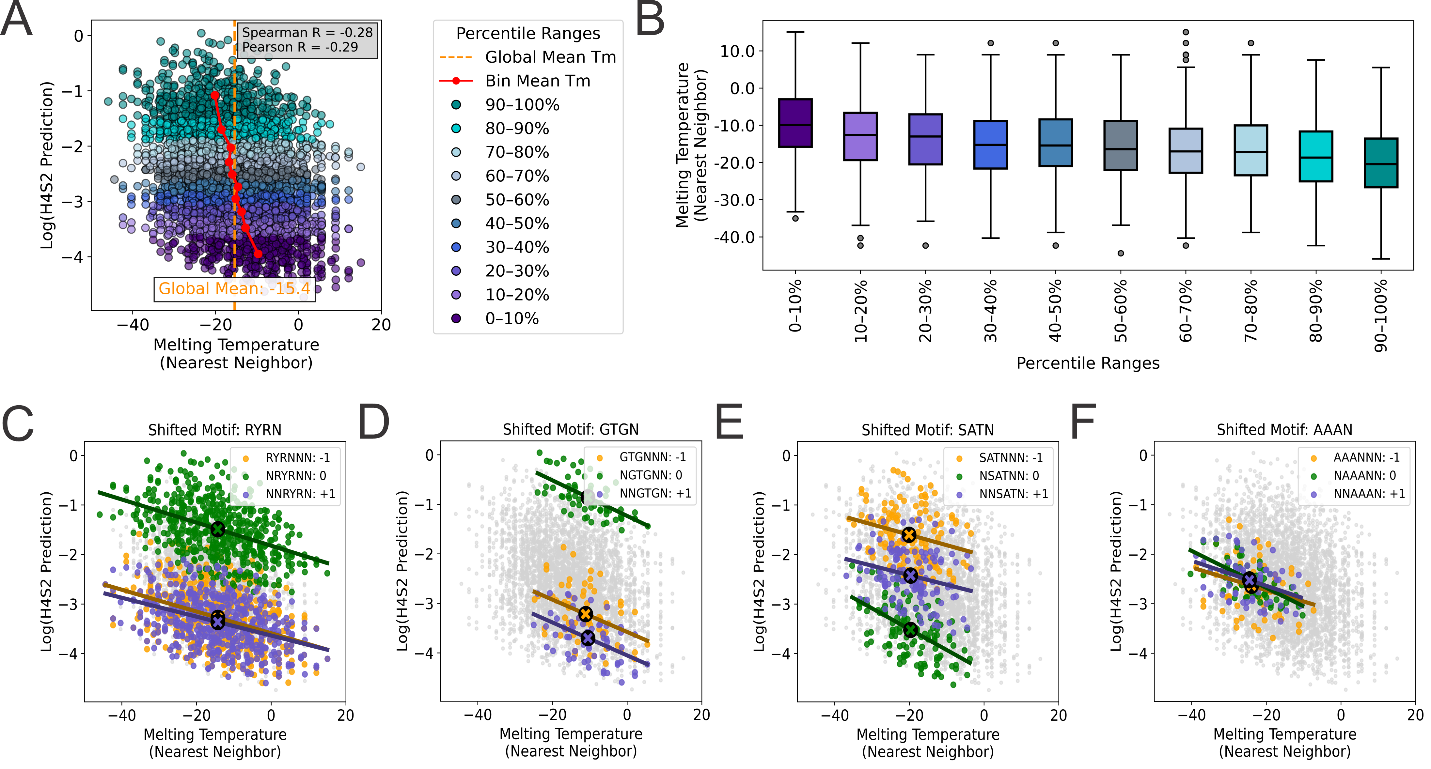


Supplementary Figure S8**. A**) A scatter plot of the log transformed H4S2 model prediction to the nearest neighbor melting temperature (Tm) calculation, with a Pearson correlation r=-0.29 and Spearman rank order correlation of (0.28 *ρ*). Every tenth percentile is binned and colored by the log transformed H4S2 model prediction. The red points show each percentile bin’s mean melting temperature **B)** A box plot of percentile bins, where the top whisker is the maxima, the bottom whisker is the minima, the top of the box is the 75^th^ percentile, the bottom of the box is the 25^th^ percentile, and together the 25^th^ to 75^th^ percentile range represents the inner quartile range. The middle line indicates the median (50^th^ percentile). **C-F)** Scatter plots of the log transformed H4S2 model prediction and the melting temperature. Each sequence motif was placed in three different sequence alignments along the H4-S2 region. **C)** Motif ‘RYRN’ placed at alignment -1 (RYRN’NN) colored in yellow, at alignment 0 (N’RYRN’N) colored in green, and at alignment +1 (NN’RYRN’) colored in lavender. The marker demarks the alignment motif’s centroid and associated darker lines correspond to the linear regression line for each alignment motif. **D)** Motif ‘GTGN’ at three alignments -1, 0 and +1 and regression results. **E)** Motif ‘SATN’ at three alignments -1, 0 and +1 and regression results. **F)** Motif ‘AAAN’ at three alignments -1, 0 and +1 and regression results. See **Figure 4** for analysis of all 4-mer sequences and **Supplemental Dataset 2** for individual regression results.


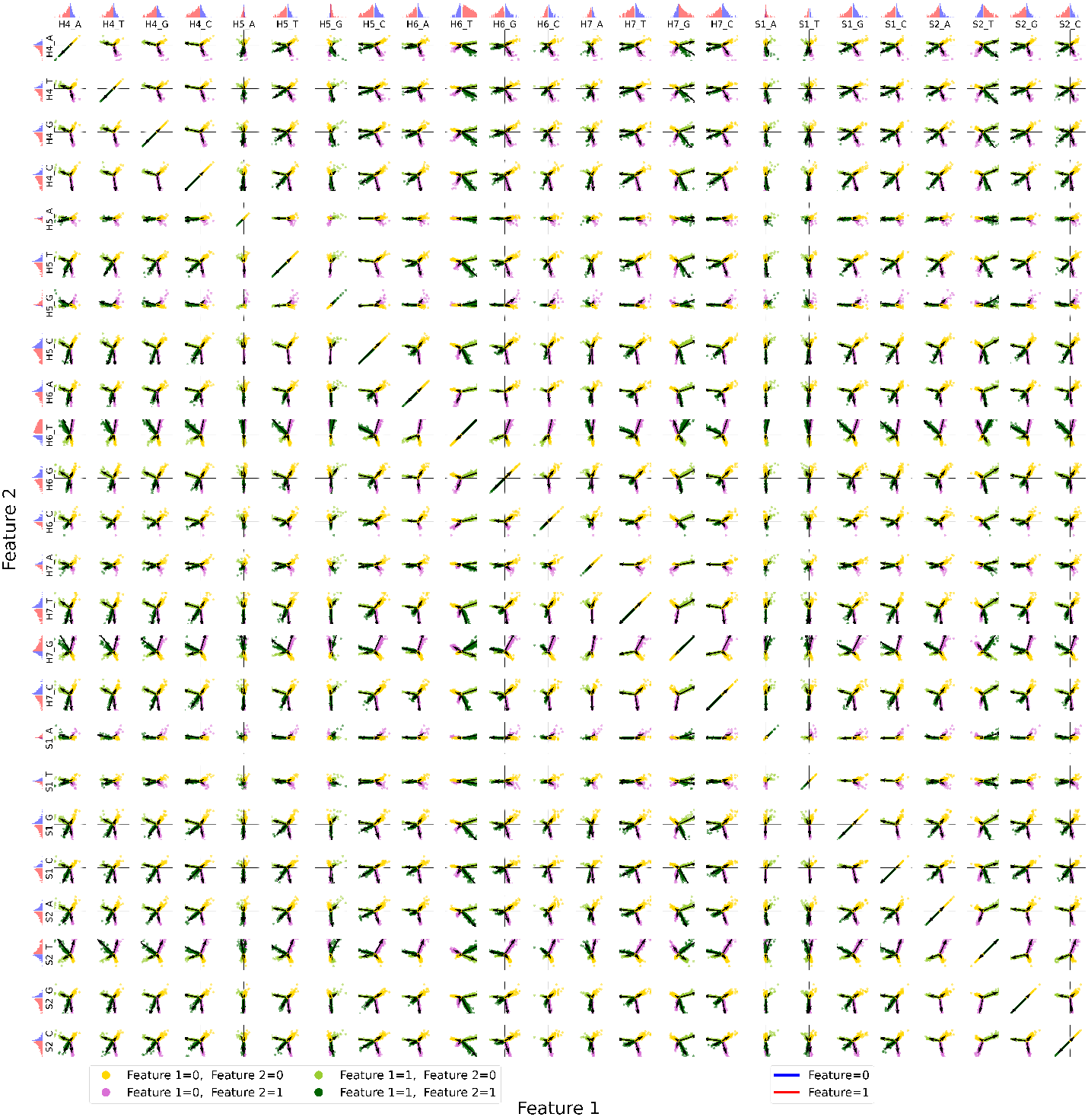


Supplementary Figure S9. Multimodal distributions of the features pairwise comparison of their SHAP values. The histograms show each feature’s SHAP value distributions, or first order interactions. The multimodal distributions modes are explained and colored by the two features’ combined features binary encodings 0,0 yellow; 0,1 pink; 1,0 light green; and 1,1 dark green. Black arrows show the cooperative relationship vectors which captures information of each mode or relationship between two feature’s combined binary feature encodings.


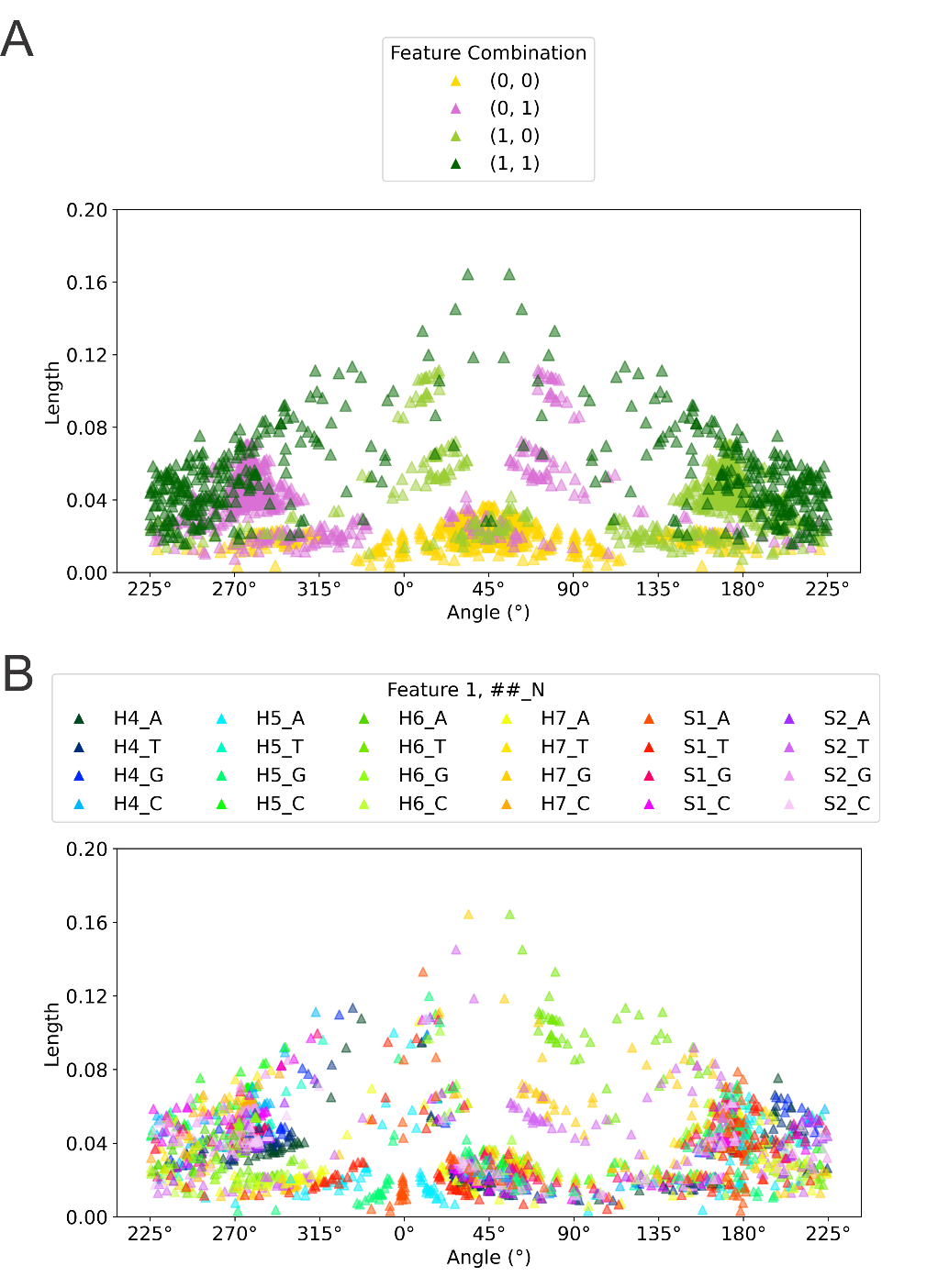


Supplementary Figure S10. Cooperative Relationship Vector (CRV) length and angle plot, generated from the pairwise comparisons of two features SHAP values and each of their 4 combinations of binary feature encodings, excluding feature self-comparisons. **A)** Colored by the combination of binary feature encodings, and **B)** colored by one feature of the two features in the pairwise comparison.


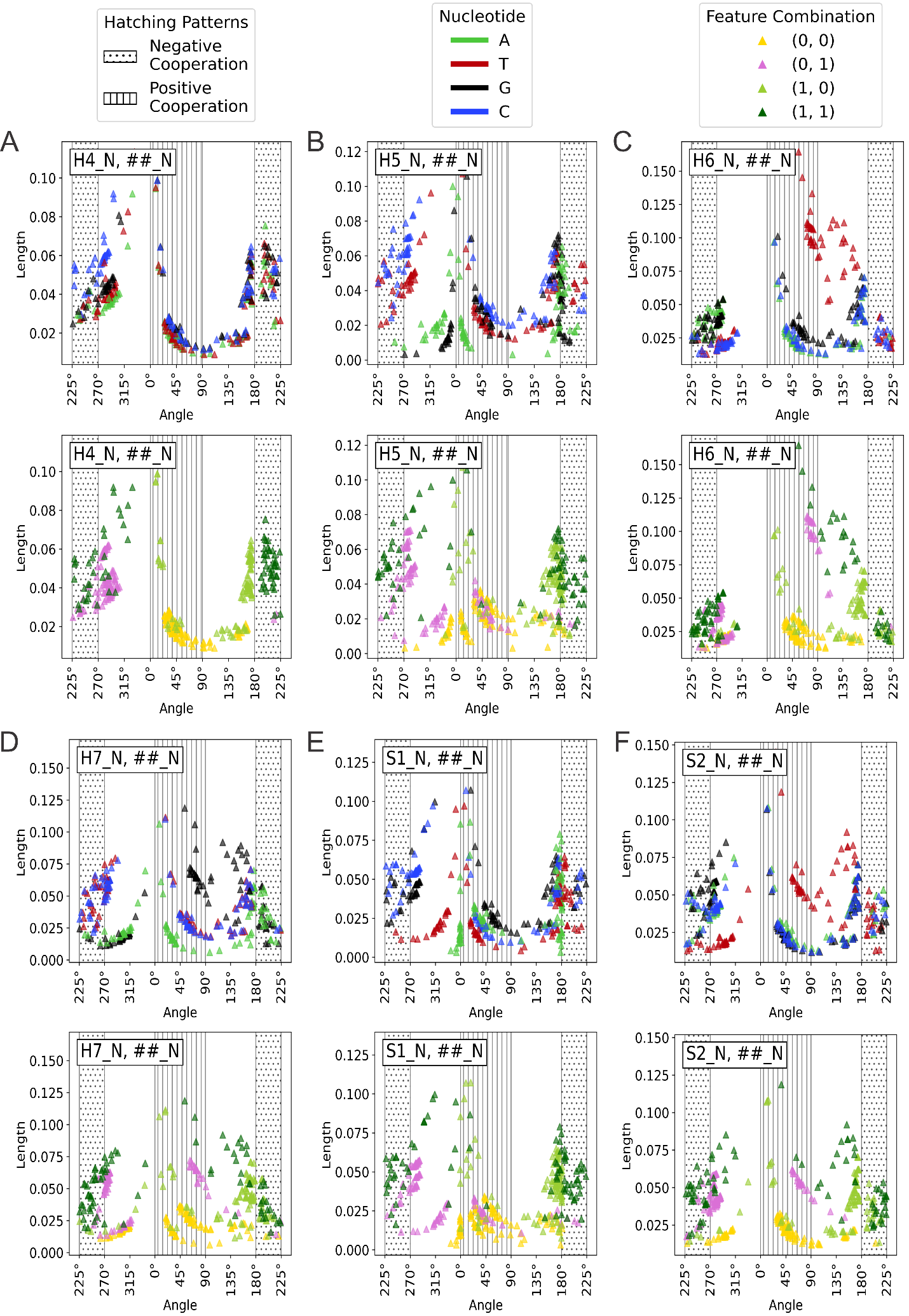


Supplementary Figure S11. Cooperative Relationship Vector (CRV) length and angle plots, isolating the cooperative relationship vectors for each position and its four nucleotide bases. The pairwise interactions nucleotides A, T, G, and C at positions **A)** H4, **B)** H5, **C)** H6, **D)** H7, **E)** S1, and **F)** S2 have with all other features. UPPER colored by nucleotide, LOWER colored by combination of binary feature encodings. **A-F)** Vertical line background highlights Q1 and cooperative relationship vectors with positive cooperation. Dotted background highlights Q3 and cooperative relationship vectors with negative cooperation.


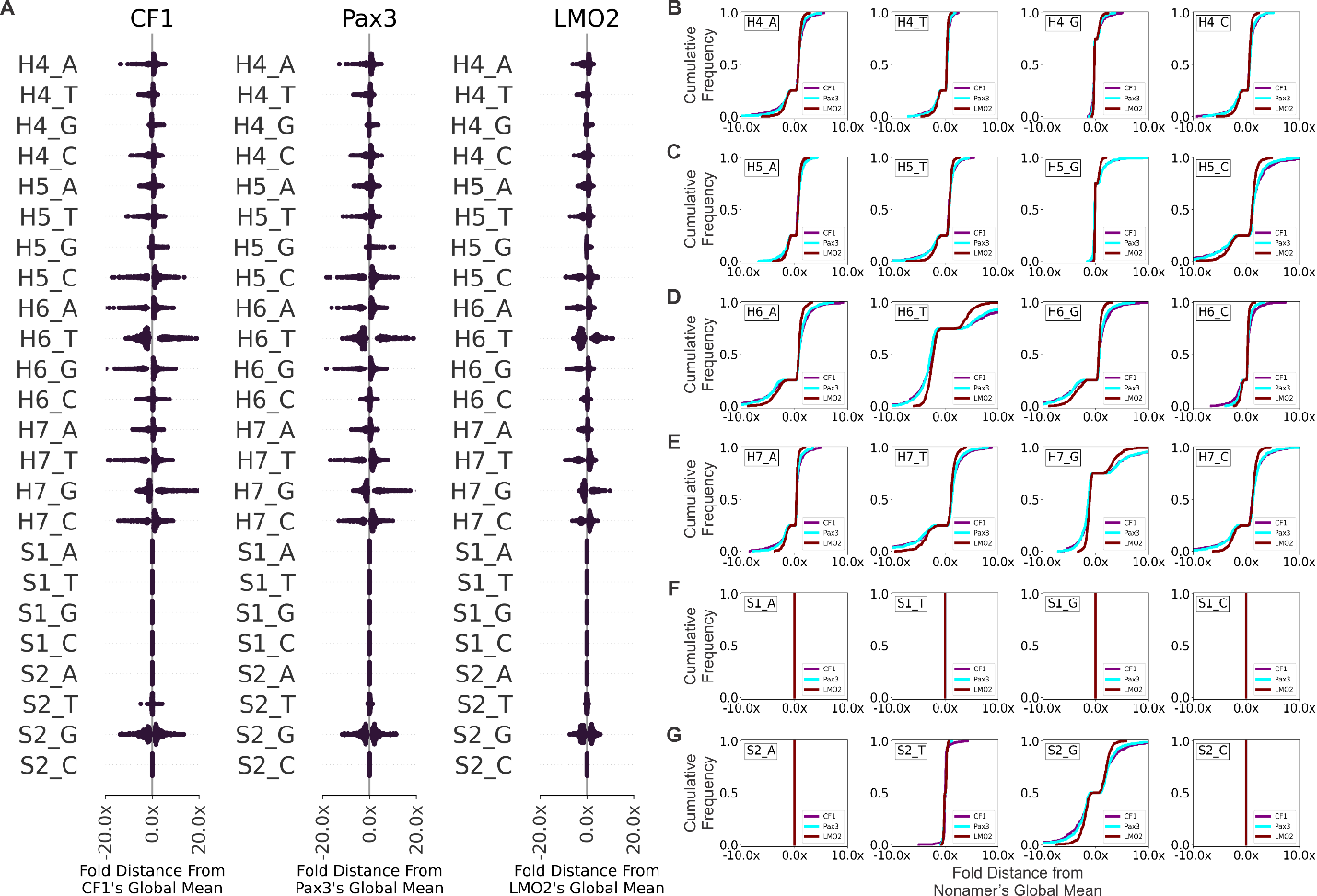


Supplementary Figure S12. **A)** The fold distance of each feature’s SHAP value distributions to the specific nonamer’s global mean. LEFT: CF1 nonamer, MIDDLE: Pax3 nonamer, RIGHT: LMO2 nonamer. **B-G)** Cumulative frequency curves of each rescaled nonamer. Plots show the cumulative frequency distribution (CFD) for positions **B)** H4, **C)** H5, **D)** H6, **E)** H7, **F)** S1, and **G)** S2.

Supplementary Table S1. Oligonucleotide sequences

| Oligo | Sequence |
| --- | --- |
| RSS 1 FWD | 5’-CGCGAGGTTTTTGTTCCAGTCTGTATCACTGTGCAGTAGG-3’ |
| RSS 1 RVS | 5’-AATTCCTACTGCACAGTGATACAGACTGGAACAAAAACCT-3’ |
| Pax3 FWD | 5’-CGCGAGGTTTTTAGTCCAGTCTGTATCACTGTGCAGTAGG-3’ |
| Pax3 RVS | 5’-AATTCCTACTGCACAGTGATACAGACTGGACTAAAAACCT-3’ |
| LMO2 FWD | 5’-CGCGATATTTTCCATCCAGTCTGTATCACTGTGCAGTAGG-3’ |
| LMO2 RVS | 5’-AATTCCTACTGCACAGTGATACAGACTGGATGGAAAATAT-3’ |
| HB-1 FWD | 5’-CGCGAGGTTTTTGTTCCAGTCTGTTAAGCAGTGCAGTAGG-3’ |
| HB-1 RVS | 5’-AATTCCTACTGCACTGCTTAACAGACTGGAACAAAAACCT-3’ |
| HB-2 FWD | 5’-CGCGAGGTTTTTGTTCCAGTCTGTAGAAGTGTGCAGTAGG-3’ |
| HB-2 RVS | 5’-AATTCCTACTGCACACTTCTACAGACTGGAACAAAAACCT-3’ |
| HB-3 FWD | 5’-CGCGAGGTTTTTGTTCCAGTCTGTGTTACAGTGCAGTAGG-3’ |
| HB-3 RVS | 5’-AATTCCTACTGCACTGTAACACAGACTGGAACAAAAACCT-3’ |
| HB-4 FWD | 5’-CGCGAGGTTTTTGTTCCAGTCTGTATCAATGTGCAGTAGG-3’ |
| HB-4 RVS | 5’-AATTCCTACTGCACATTGATACAGACTGGAACAAAAACCT-3’ |
| S10-N4_B1 FWD | 5’-CGCGAGGTTTCTTCCTCAGTCTGTATCACTGTGCAGTAGG-3’ |
| S10-N4_B1 RVS | 5’-AATTCCTACTGCACAGTGATACAGACTGAGGAAGAAACCT-3’ |
| S10-N4_B2 FWD | 5’-CGCGAGGTTTCCATTGTAGTCTGTATCACTGTGCAGTAGG-3’ |
| S10-N4_B2 RVS | 5’-AATTCCTACTGCACAGTGATACAGACTACAATGGAAACCT-3’ |
| S10-N4_B3 FWD | 5’-CGCGAGGTTTGAAATTCAGTCTGTATCACTGTGCAGTAGG-3’ |
| S10-N4_B3 RVS | 5’-AATTCCTACTGCACAGTGATACAGACTGAATTTCAAACCT-3’ |
| S10-N4_B4 FWD | 5’-CGCGAGGTTTATGAATTAGTCTGTATCACTGTGCAGTAGG-3’ |
| S10-N4_B4 RVS | 5’-AATTCCTACTGCACAGTGATACAGACTAATTCATAAACCT-3’ |

REFERENCES

1. Jovel, J. and Greiner, R. (2021) An Introduction to Machine Learning Approaches for Biomedical Research. *Front Med (Lausanne)*, **8**, 771607.

2. Gupta, Y.M., Kirana, S.N. and Homchan, S. (2024) Representing DNA for machine learning algorithms: A primer on one-hot, binary, and integer encodings. *Biochem Mol Biol Educ*, **53**, 142-146.

3. Jayalakshmi, T. and Santhakumaran, A. (2011) Statistical normalization and back propagation for classification. *International Journal of Computer Theory and Engineering*, **3**, 1793-8201.

4. Zeiler, M.D. and Fergus, R. (2014) Visualizing and Understanding Convolutional Networks. *In Computer Vision—ECCV 2014*, 818-833.

5. Fan, F.L., Xiong, J., Li, M. and Wang, G. (2021) On Interpretability of Artificial Neural Networks: A Survey. *IEEE Trans Radiat Plasma Med Sci*, **5**, 741-760.

6. Wojciuk, M., Swiderska-Chadaj, Z., Siwek, K. and Gertych, A. (2024) Improving classification accuracy of fine-tuned CNN models: Impact of hyperparameter optimization. *Heliyon*, **10**, e26586.

7. Bischl, B., Binder, M., Lang, M., Pielok, T., Richter, J., Coors, S., Thomas, J., Ullmann, T., Becker, M., Boulesteix, A.-L. *et al.* (2023) Hyperparameter optimization: Foundations, algorithms, best practices, and open challenges. *WIREs Data Mining and Knowledge Discovery*, **13**, e1484.

8. Le Cun, Y. (1988) A Theoretical Framework for Back-Propagation. *Proceedings of the 1988 Connectionist Models Summer School, CMU, Pittsburg, PA*, 21-28.

9. Srivastava, N., Hinton, G., Krizhevsky, A., Sutskever, I. and Salakhutdinov, R. (2014) Dropout: A Simple Way to Prevent Neural Networks from Overfitting. *Journal of Machine Learning Research*, **15**, 1929-1958.
